# DEAD-box protein p68 is regulated by β-catenin/transcription factor 4 to maintain a positive feedback loop in control of breast cancer progression

**DOI:** 10.1186/s13058-014-0496-5

**Published:** 2014-12-12

**Authors:** Kiran Kumar Naidu Guturi, Moumita Sarkar, Arijit Bhowmik, Nilanjana Das, Mrinal Kanti Ghosh

**Affiliations:** Signal Transduction in Cancer and Stem Cells Laboratory, Cancer Biology and Inflammatory Disorder Division, Council of Scientific and Industrial Research-Indian Institute of Chemical Biology (CSIR-IICB), 4 Raja S C Mullick Road, Jadavpur, Kolkata 700032 India

## Abstract

**Introduction:**

Nuclear accumulation of β-catenin is important for cancer development and it is found to overlap with p68 (DDX5) immunoreactivity in most breast cancers, as indicated by both clinical investigations and studies in cell lines. In this study, we aim to investigate the regulation of p68 gene expression through β-catenin/transcription factor 4 (TCF4) signaling in breast cancer.

**Methods:**

Formalin-fixed paraffin-embedded sections derived from normal human breast and breast cancer samples were used for immunohistochemical analysis. Protein and mRNA expressions were determined by immunoblotting and quantitative RT-PCR respectively. Promoter activity of p68 was checked using luciferase assay. Occupancy of several factors on the p68 promoter was evaluated using chromatin immunoprecipitation. Finally, a syngeneic mouse model of breast cancer was used to assess physiological significance.

**Results:**

We demonstrated that β-catenin can directly induce transcription of p68 promoter or indirectly through regulation of c-Myc in both human and mouse breast cancer cells. Moreover, by chromatin immunoprecipitation assay, we have found that both β-catenin and TCF4 occupy the endogenous p68 promoter, which is further enhanced by Wnt signaling. Furthermore, we have also established a positive feedback regulation for the expression of TCF4 by p68. To the best of our knowledge, this is the first report on β-catenin/TCF4-mediated p68 gene regulation, which plays an important role in epithelial to mesenchymal transition, as shown *in vitro* in breast cancer cell lines and *in vivo* in an animal breast tumour model.

**Conclusions:**

Our findings indicate that Wnt/β-catenin signaling plays an important role in breast cancer progression through p68 upregulation.

**Electronic supplementary material:**

The online version of this article (doi:10.1186/s13058-014-0496-5) contains supplementary material, which is available to authorized users.

## Introduction

Compelling evidences indicate that the Wnt/β-catenin signaling is implicated in different stages of mammary gland development and is also important for mammary oncogenesis when aberrantly activated [1]-[5]. Genetic mutations in adenomatous polyposis coli (APC) and catenin (cadherin-associated protein) beta 1 (CTNNB1), the components of the Wnt/β-catenin signaling pathway, are the major contributors of colorectal cancer although they are typically not the key factors associated with breast cancer. It has been demonstrated that only 6% of breast tumours contain mutations in the APC gene but no mutations were detected in CTNNB1 [6],[7]. However, Wnt proteins (1, 3a, 4, 5a, 7b, 10b and 14) [8]-[10] and multiple Frizzled receptors (Fzd4/7) are reported to be overexpressed in human breast cancer cell lines and primary tumours [11],[12]. Recently, it has been documented that low-density lipoprotein-related protein (LRP)6 but not LRP5 is frequently upregulated in a subset of human breast carcinomas and downregulation of LRP6 is sufficient to inhibit breast cancer tumourigenesis [13]. Moreover, Dishevelled 1 (DVL1), a central regulator of Wnt signaling is found to be upregulated in breast cancer [14]. In addition to this, epigenetic silencing of the Wnt antagonists secreted Frizzled-related proteins (sFRPs) and Wnt inhibitory factor-1 (WIF-1) leads to aberrant regulation of Wnt/β-catenin signaling in both primary breast tumours and cell lines [15]-[17]. Again, approximately 60% of primary breast tumours show cytoplasmic or nuclear accumulation of β-catenin rather than its membrane localization, and this is correlated with poor prognosis [18].

p68 was first discovered through its immunological cross-reactivity with the anti-SV40 large T monoclonal antibody [19]. Molecular similarity of p68 (an ATP-dependent RNA helicase) with both the large T antigen and eIF-4A (an ATP-dependent DNA helicase) implied that p68 may function as both RNA and DNA helicase [20]. Moreover, p68 knockout mice are embryonically lethal (E11.5), indicating its importance in the development process [21]. p68 was shown to bind, unwind and rearrange RNA secondary structures and it is also a crucial factor in the processing, alternate splicing and degradation of mRNA [22]-[24]. Subsequently, p68 has been implicated in a wide range of biological processes, and early studies of this protein indicate its possible involvement in the regulation of proliferation and organ differentiation [22]. Recently, p68 has been demonstrated to act as a potent transcriptional co-activator of estrogen receptor [25],[26], androgen receptor [27], tumour suppressor p53 [28], MyoD [29] and β-catenin [30]. p68’s activation as a result of its phosphorylation at Tyr^593^ by platelet-derived growth factor (PDGF) was shown to be associated with cellular transformation and epithelial to mesenchymal transition (EMT) in colon cancer by promoting nuclear translocation of β-catenin, and upregulation of its target genes like cyclin D1 and c-myc [31],[32]. In addition to this, modification of p68 by the small ubiquitin-like modifier SUMO-2 was found to modulate its activity as a transcriptional regulator, favouring repression [33]. It has been found that p68 is constitutively overexpressed in various cancers like colon [34], breast [35], prostate [27], head and neck as well as cutaneous squamous cell carcinoma [36]. Serum-induced p68 expression in Swiss 3 T3 fibroblasts is involved in cellular proliferation and also connected to organ differentiation and maturation of the foetus [37].

p68 regulates the expression of several oncogenes through co-activation of β-catenin-mediated transcription, and controls tumour growth and metastasis. Although, there is an expansive evidence of literature deciphering the central role of p68 with respect to β-catenin in the architecture of intracellular signaling networks, little is known about its transcriptional regulation that may contribute to cancer development. Moreover, cellular consequences due to the modulation of its expression are not yet completely understood. Such knowledge might provide invaluable insights into the molecular mechanisms with respect to p68 in the context of oncogenesis.

## Methods

### Cell culture, transfection and drug treatments

HEK293T (human embryonic kidney); MCF7, MDA-MB 231 (human breast cancer); 4T1 (mouse breast cancer); H1299 (lung adenocarcinoma) and HCT116 (colon cancer) cell lines used in this study were obtained from the American Type Culture Collection (ATCC). Cell culture and transfections were performed using standard procedure as described previously [38]. Small interfering RNAs (siRNAs) were purchased from Sigma-Aldrich (St Louis, MO, USA) (β-catenin), Cell Signaling Technology (Beverly, MA, USA) (p68) and Santa Cruz Biotechnology (Santa Cruz, CA, USA) (tcf4 and c-myc). G418 (2 mg/ml)-resistant MCF7 stable cells were selected by homogeneous colony formation as described earlier [39].

### Wnt3A conditioned medium

Wnt3a-L cells were split at the ratio of 1:10 in 10 ml medium using standard procedure. Medium was collected from two consecutive batches of cells cultured for four days. Wnt-3a conditioned medium (Wnt3a-CM) was either freshly used with serum-free media (1:1) or stored at 4°C until further use.

### Expression plasmids

The human wnt3a gene was cloned in pcDNA4/TO and further sub-cloned into pcDNA3.1-myc-his. Cloning of c-myc in pcDNA3.1-myc-his was done previously [40]. pEGFP-c1-β-catenin and pEGFP-c1-p68 were sub-cloned from pGZdx-β-catenin and pGS5-p68 respectively. Human and mouse p68 promoters (-1200 to +200 and -1200 to +175 respectively) were cloned in pGL3-basic plasmid. Point mutations in these p68 promoters were generated by Site-Directed Mutagenesis using QuikChange XL kit (Stratagene, San Diego, CA, USA). Transcription factor 4 (TCF4) promoter (-1001 to +303) was cloned into pGL3-basic plasmid. All the constructs were verified by restriction digestions and confirmed by sequencing. Sequences of all the primers used are available in Table S1 in Additional file 1.

### Immunocytochemistry (ICC)

For ICC, cells were processed as described earlier [41]. Briefly, after permeabilization, cells were incubated with primary antibodies (1:200) overnight at 4°C followed by incubation with Alexa Fluor (488 or 594)-tagged secondary antibodies (1:500) (Molecular Probes, Eugene, OR, USA). Primary antibodies (β-catenin and TCF4) were purchased from Cell Signaling Technology. Images were taken with BX61 upright fluorescence microscope (Olympus, Center Valley, PA, USA) using Image-Pro Plus software (Media Cybernetics, Silver Spring, MD, USA).

### Immunoblotting (IB)

Preparation of whole cell lysates (WCL), cytoplasmic and nuclear extracts and IB were performed as described previously [41]. Either GAPDH or β-actin was used as a loading control. Primary antibodies were purchased from Abcam (Cambridge, UK) (p68), Cell Signaling Technology (β-catenin, Cyclin D1, c-Myc, TCF4, Myc-Tag, E-cadherin, N-cadherin and Vimentin) and Santa Cruz Biotechnology (GAPDH, α-Tubulin, LaminB, β-actin and TCF4). Horseradish peroxidase (HRP)-tagged anti-rabbit and anti-goat secondary antibodies used were from Cell Signaling Technology and Sigma-Aldrich respectively.

### Quantitative RT-PCR (qRT-PCR)

Total RNA was extracted and converted to cDNA, which was subsequently used for qRT-PCR analysis [40] using Power SYBR Green Master Mix on a 7500 Fast Real-Time PCR system (Applied Biosystems, Carlsbad, CA, USA). 18S rRNA served as the internal control. Sequences of all the primers used in qRT-PCR are given in Table S1 in Additional file 1. Standard deviation (SD) calculations are based on three technical replicates of two independent biological repeats.

### Luciferase assays

Cells were transiently transfected with pGL3-p68 promoter luciferase reporter construct(s) along with Renilla luciferase plasmid (pRL-TK) and subjected to various treatments as indicated in the relevant figure(s). Luciferase activity was determined by luminometry using the GLOMAX 20/20 luminometer (Promega, Madison, WI, USA) by the dual-luciferase assay system (Promega), as specified by the manufacturer. Quantification was based on three technical replicates over at least two independent biological repeats.

### Chromatin immunoprecipitation (ChIP)

Sonicated cross-linked chromatin fragments were prepared from cells treated with formaldehyde as described earlier [41],[42]. Briefly, equal amounts of all pre-cleared chromatin fragments (250 μg) were incubated with primary antibodies for 12 h at 4°C followed by pull down with 3% bovine serum albumin (BSA)-blocked protein G sepharose beads. Primary antibodies against β-catenin, TCF4, c-Myc and normal rabbit immunoglobulin G (IgG) were purchased from Cell Signaling Technology. Purified DNAs from immunoprecipitated chromatin fragments were used in PCR reactions with a standard programme using Qiagen’s Top Taq master mix (Qiagen, Venlo, Netherlands). The PCR products were analysed in 2% agarose gel. Quantitative RT-PCR (qRT-PCR) reactions were performed using SYBR Green master mix. All the primers used are listed in Table S1 in Additional file 1.

### Soft agar colony formation assay and invasion assay

Colony formation assays in soft agar were performed in triplicate as described earlier [43]. Images were captured by Olympus IX81 microscope using Image-Pro Plus software (Olympus) at 100× optical magnification and colony-forming efficiency was quantified. Matrigel invasion assay was performed as described previously [44]. Images were captured by Olympus IX81 microscope at 40X magnification. Statistical analysis was performed by Student’s *t* test using GraphPad software with level of significance *P* <0.001 (GraphPad Software, San Diego, CA, USA).

### Immunohistochemistry (IHC)

Formalin-fixed paraffin-embedded (FFPE) tissue sections derived from normal human breast (n = 10) and breast cancer samples (n = 20) were obtained from Indian patients after formal approval from ethical committee of both Council of Scientific and Industrial Research (CSIR)-Indian Institute of Chemical Biology (IICB) and Park Clinic (registered under WB Societies Act. 1961). All patients involved in the study agreed to participate and publish the research outcome. IHC was performed and H-scores were calculated as described previously [45]. Primary antibodies were from Abcam (p68 and Snail), Cell Signaling Technology (TCF4, E-cadherin, proliferating cell nuclear antigen (PCNA) and Vimentin) and Santa Cruz Biotechnology (β-catenin).

### Orthotopic syngeneic mouse model of breast cancer

All animal care and experimentation conformed to the Committee for the Purpose of Control and Supervision of Experiments on Animals (CPCSEA) (Govt. of India) following internationally recognized guidelines. The animal ethics approval was granted by ‘IICB-Animal Ethics Committee (IICB-AEC), CSIR, Govt. of India’. To generate the tumour model, three- to four-week-old BALB/c mice were injected once with either EV or p68 knockdown 4T1 stable cells (1 × 10^5^) in the mammary fat pad region. Growth of the tumour was observed for a period of 21 days after which the mice were sacrificed and the tumours were excised, fixed in 10% buffered formalin and embedded in paraffin for further IHC analysis.

### Statistical analysis and densitometry

All statistical calculations were done using GraphPad QuickCalcs calculator, [46]. For the analysis of statistical significance of the H scores, *U* test or Student’s *t* test (unpaired) was used. In all the experiments a value of *P* <0.05 was considered as statistically significant. Image quantification and densitometric scanning of immunoblots were done using Image J software (Bethesda, MD, USA).

## Results

### Canonical Wnt signaling regulates p68 expression

Canonical Wnt signaling in human colon and breast cancer cells plays a key role for the aberrant activation of the β-catenin/TCF4 in tumour progression. Like β-catenin, p68 is an important transcriptional regulator, crucial for early growth and development and is also associated with cell proliferation [37],[47]. Overexpression of p68 is common in most of the human tumours including breast [30]. Here, we are interested in investigating the possible connections among these intensively studied oncogenes.

**Figure 1:**
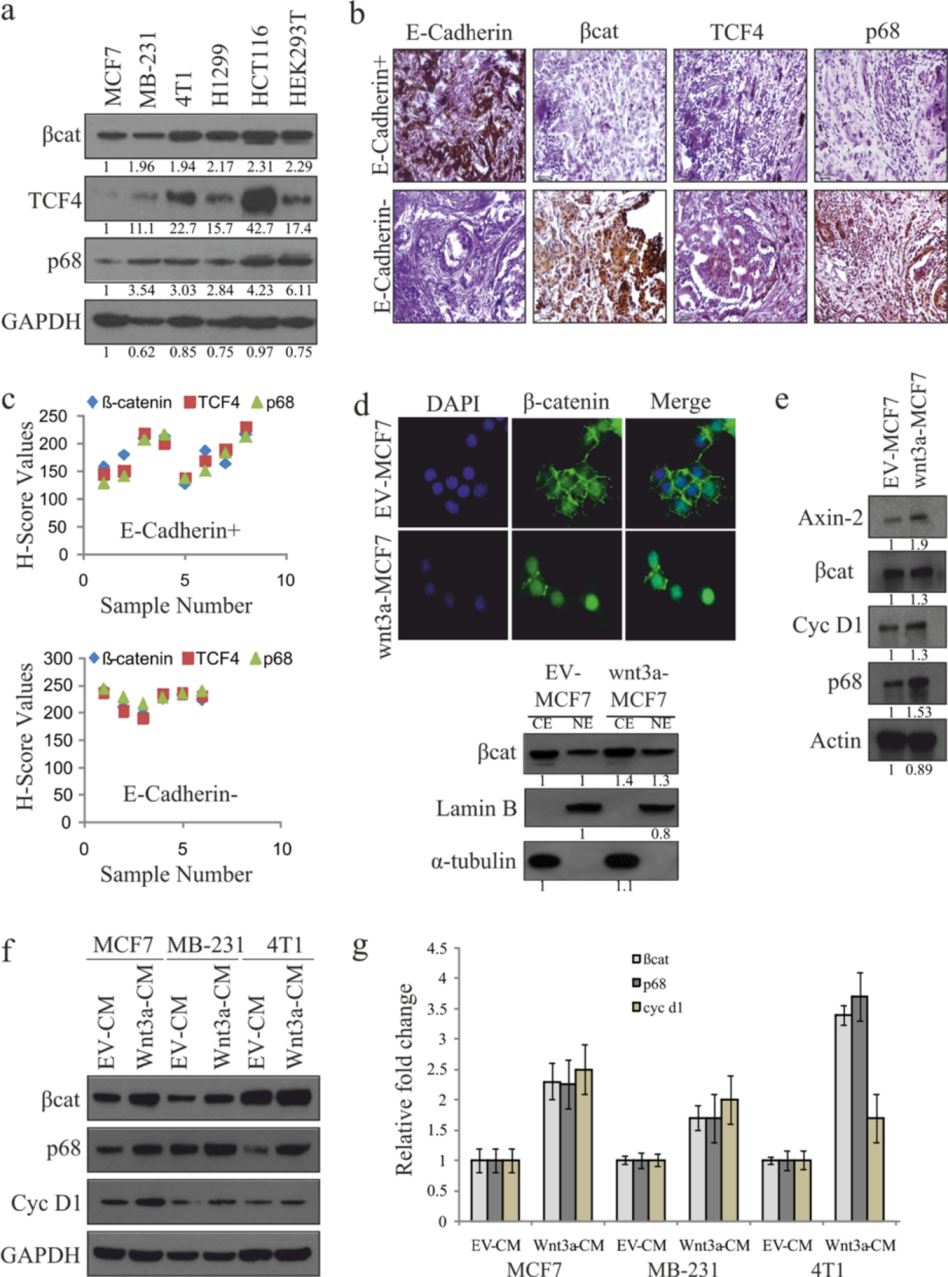
**Canonical Wnt signaling upregulates p68 expression. (a)** Whole cell lysates (WCLs) were prepared from MCF7, MDA-MB 231, 4T1, H1299, HCT116 and HEK293T cells. β-catenin, transcription factor 4 (TCF4) and p68 protein levels were analysed by immunoblotting (IB). Densitometry values are given below the blots. **(b, c)** Breast cancer patient samples (E-cadherin^+^ and E-cadherin^-^) were analysed by immunohistochemistry (IHC) using antibodies against β-catenin, TCF4 and p68, as well as E-cadherin, to differentiate the samples. H scores of these samples were determined. **(d)** Wnt3a-MCF7 and empty vector (EV)-MCF7 stable cells were immunostained with β-catenin (primary) and Alexa Fluor488 (secondary, Green) antibodies and observed under fluorescence microscope to see its localisation pattern. Images were captured along with DAPI-stained nuclei at 600X magnification (scale: 10 μm) (top). Cytoplasmic and nuclear extracts were prepared from Wnt3a-MCF7 and EV-MCF7 stable cells and were analysed for β-catenin by IB. Densitometry values are given below the blots (bottom). **(e)** WCLs of Wnt3a-MCF7 and EV-MCF7 stable cells were analysed for Axin-2, β-catenin, Cyclin D1 and p68 proteins by IB. Densitometry values are given below the blots. **(f, g)** MCF7, MDA-MB 231 and 4T1 cells were serum starved for 24 h before treatment with Wnt3a condition medium (Wnt3a-CM). WCLs and total RNAs were prepared from 24 h post-treated cells to check the expression of β-catenin, p68 and Cyclin D1 by IB and qRT-PCR.

To address this issue, we first examined the expression of β-catenin, TCF4 and p68 in various established cancer cell lines and HEK293T cells (Figure 1a). We have also analysed the expression pattern of these molecules by IHC analysis in human breast tumour and normal samples (Figure 1b and Figure S1 in Additional file 2) and classified based on the E-cadherin expression status [48]-[50]. The results indicate that there is a probable correlation for β-catenin and TCF4 with p68 in the cancer cell lines. Moreover, this positive correlation was also found in E-cadherin null samples (Figures 1b and c). Our results also indicate that p68 expression is quite low in MCF-7 cells as compared to highly metastatic MDA-MB 231 and mouse 4T1 cells with reduced expression or absence of E-cadherin, which is corroborated with the previous findings in breast tumours and cell lines [35]. Since Wnt3a is known to stabilize β-catenin and increase its nuclear accumulation, we generated constitutively expressing Wnt3a stable MCF7 (Wnt3a-MCF7) cells and examined the cytoplasmic/nuclear distribution of β-catenin in these cells under serum-starved conditions. Here, we visualised the localisation pattern of β-catenin by both immunofluorescence and IB and observed an increased level of β-catenin in the nucleus of these cells when compared to the empty vector- containing control (EV-MCF7) cells (Figure 1d). Wnt3a-MCF7 cells showed a significant increase in p68 level along with β-catenin and its targets Cyclin D1 and Axin-2 (positive control) compared to EV-MCF7 (Figure 1e). To strengthen our finding, we have analysed p68 expression in various breast cancer cell lines under enhanced Wnt signaling by Wnt3a-CM treatment. We observed that Wnt3a-CM upregulates p68 expression at both protein and mRNA level (Figures 1f and g), where Cyclin D1 was kept as a positive control. We have further observed a similar effect in HEK293T and other cancer cell lines (Figure S2 in Additional file 3). Further, we observed that the stabilized β-catenin due to GSK3β inactivation in lithium chloride (LiCl)-treated serum-starved MCF7 cells, leads to increased expression of p68, where Cyclin D1 served as positive control (Figure S3 in Additional file 4). Collectively, all these results clearly indicate that p68 expression is controlled by Wnt/β-catenin signaling.

### β-catenin and TCF4 control p68 transcription

**Figure 2:**
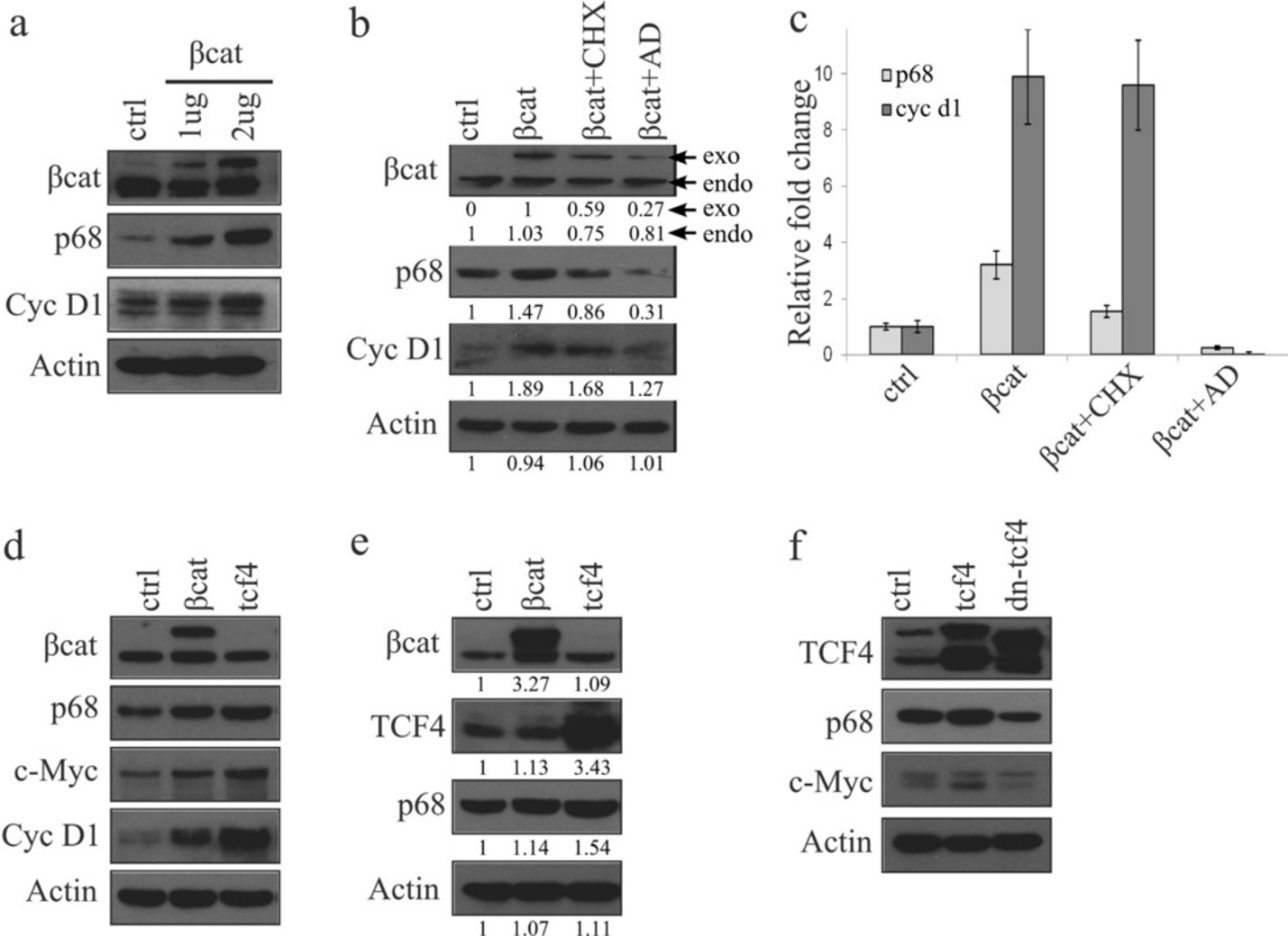
**β-catenin/TCF4 is directly involved in p68 expression. (a)** MCF7 cells were transfected with 1 and 2 μg of pEGFP-β-catenin. Expressions of p68 and β-catenin target Cyclin D1 were analysed by immunoblotting (IB) with whole cell lysates (WCLs) prepared from 36 h post-transfected cells. **(b, c)** MCF7 cells transfected with pEGFP-β-catenin. After 36 h, cells were kept in the presence of either translational (CHX) or transcriptional (AD: actinomycin D) inhibitors for another 6 h and analysed by IB and qRT-PCR for expression of p68 and cycinD1 genes. Densitometry values are given below the blots for IB. Exo and endo refers to the exogenous and endogenous band of β-catenin respectively. **(d, e)** 4T1 and HEK293T cells were transfected with either β-catenin or tcf4. WCLs prepared from 36 h post-transfected cells and expressions of target genes, as shown in the figure, were determined by IB using respective antibodies. Densitometry values are given below the blots. **(f)** HEK293T cells were transfected with either WT-TCF4 or DN-TCF4, and expressions of p68 and β-catenin target cMyc were analysed by IB with WCLs prepared from 36 h post-transfected cells.

It was interesting for us to discern the role of β-catenin in the regulation of the p68 gene expression. For this, we transiently overexpressed β-catenin in MCF7 cells and examined its effect on p68 expression, which increased with enhanced expression of β-catenin in a dose-dependent manner (Figure 2a). Again, to understand whether this regulation is a transcriptional or post-translational event, we overexpressed β-catenin in the presence of either cycloheximide (CHX, translational inhibitors) or actinomycin D (AD, transcriptional blocker). In the presence of overexpressed β-catenin, increased expression of p68 was drastically diminished by transcriptional inhibition rather than translational inhibition (Figures 2b and c).

To further support these results we have investigated this β-catenin/TCF4-mediated p68 gene regulation in both mouse 4T1 and human HEK293T cells. Here, we observed that overexpression of either β-catenin or TCF4 leads to increased expression of p68 along with Wnt targets c-Myc and Cyclin D1 (Figures 2d and e). Conversely, p68 expression was reduced in HEK293T cells transiently overexpressing dominant negative TCF4 (DN-TCF4) (Figure 2f). We have also observed p68 mRNA upregulation in TCF4 overexpressing HEK293T cells (Figure S4 in Additional file 5).

Hence, it can be stated that the transcriptional upregulation of p68 by ectopic expression of β-catenin or TCF4 indicates a direct involvement of the β-catenin/TCF4 transcription complex on p68 gene expression.

### Wnt/β-catenin target c-Myc additionally contributes to p68 gene expression

**Figure 3:**
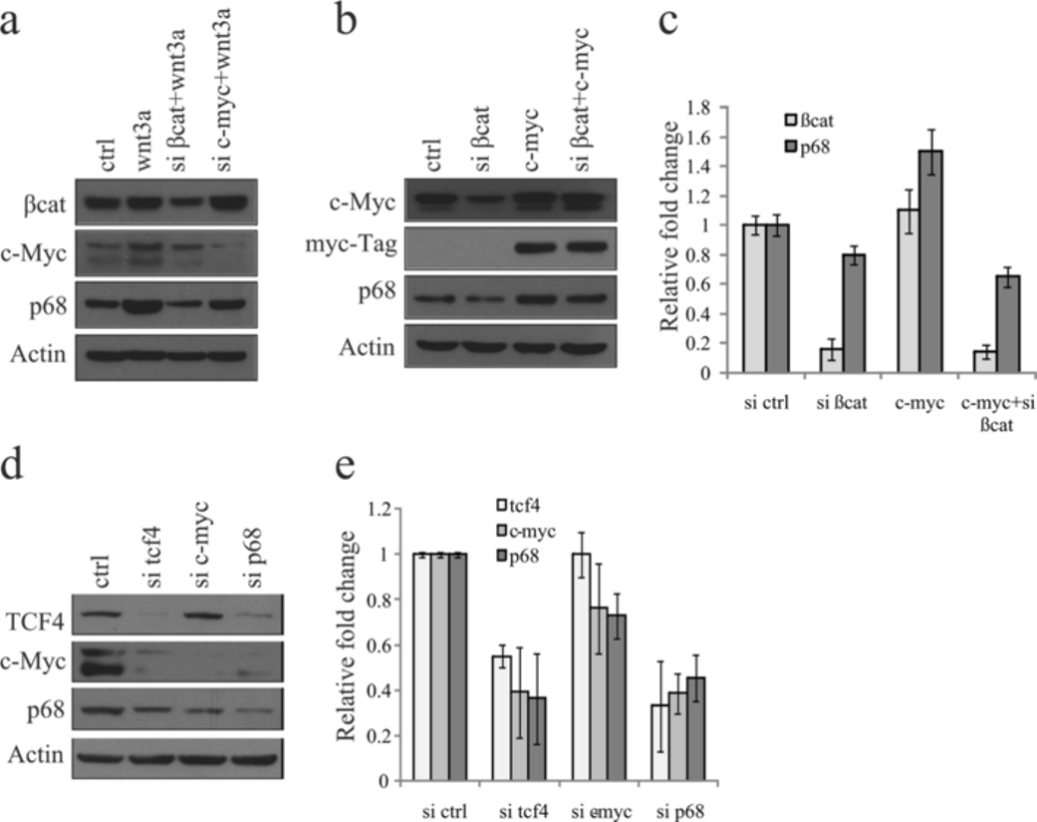
**p68 gene expression is controlled by both β-catenin and c-Myc. (a)** HEK293T cells were transfected with small interfering RNAs (siRNAs) targeting either β-catenin or c-Myc. Scrambled siRNA-transfected cells were kept as control. After 24 h, cells were transfected with wnt3a and kept for another 24 h. Whole cell lysates (WCLs) were prepared and proteins were analysed by immunoblotting (IB) as shown in the figure. **(b, c)** β-catenin was knocked down in presence or absence of c-Myc overexpression by ectopic expression of myc-tagged c-Myc in H1299 cells. WCLs and total RNAs of 36 h post-transfected cells were analysed for p68 expression by IB and qRT-PCR. **(d, e)** 4T1 cells were transfected with siRNA targeting transcription factor 4 (TCF4), c-Myc, p68 or scrambled siRNA independently, and expression of respective proteins and mRNAs were analysed by IB and qRT-PCR after 36 h of transfection.

To decipher the significance of c-Myc in the Wnt signaling-mediated tumourigenesis and its involvement in p68 gene expression, if any, we have knocked down either β-catenin or c-Myc in HEK293T cells followed by transiently overexpressing Wnt3a. We have observed that the resultant knockdown of either β-catenin or c-Myc reduces the Wnt3a-dependent increase in the expression of p68 as well as Wnt/β-catenin target c-Myc (Figure 3a). We have also overexpressed either c-Myc or β-catenin in HEK293T cells. We observed increased expression of p68 without additional signaling by wnt3a overexpression (Figure S5 in Additional file 6). We have further confirmed this regulation by knocking down β-catenin in c-Myc-overexpressed H1299 cells and analysed by IB and qRT-PCR (Figures 3b and c). Consistently, upon knockdown of either TCF4, c-Myc or p68 in mouse 4T1 cells using respective siRNAs, we have found a substantial decrease in the expression levels of p68 (Figures 3d and e). Surprisingly, here we have also observed an unexpected result in p68 knockdown cells where TCF4 was abruptly diminished indicating a relation between them. All these results suggest that both β-catenin/TCF4 and c-Myc are important factors in regulating p68 gene expression.

### Enhanced expression of both p68 and TCF4 by Wnt/β-catenin signaling constitutes a positive feedback loop in breast cancer cells

A positive feedback loop exists to regulate TCF4 expression mediated by β-catenin/p300 in the endometrial carcinoma (Em Ca) cells [51]. Since p68 is the co-activator of β-catenin, we sought to analyse the importance of p68 in this type of feedback mechanism involving TCF4 expression. To examine this, we have selected MCF7 cells where both TCF4 and p68 expressions are low compared to other cancer cell lines.

**Figure 4:**
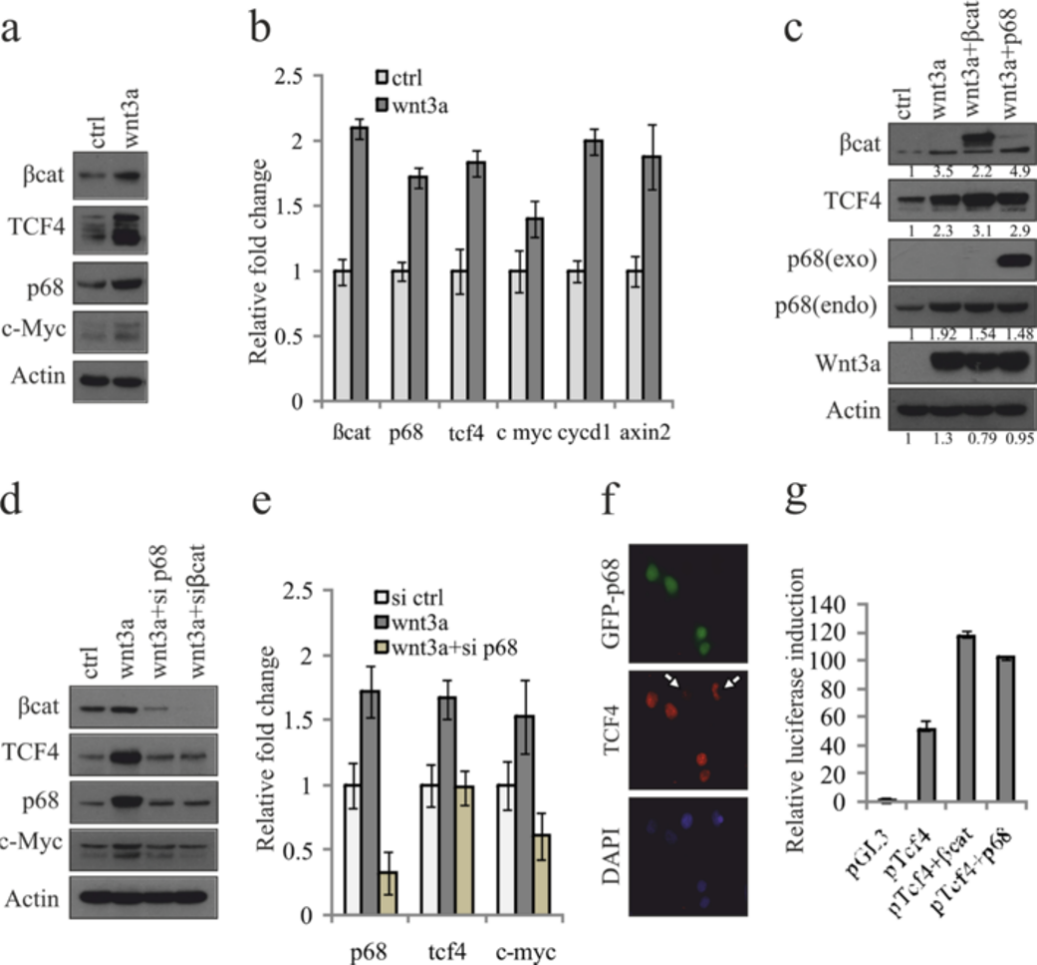
**p68 co-activates β-catenin-mediated TCF4 gene expression.** To examine this Wnt3a expressing stable MCF7 cells (wnt3a) were prepared and used where empty vector (EV)-MCF7 cells (ctrl) kept as control. **(a, b)** Stable MCF7 cells were serum starved for 48 h and analysed for expression of various transcription factor 4 (TCF4) target genes by immunoblotting (IB) and qRT-PCR. **(c, d)** p68 and β-catenin were either overexpressed (c) or knocked down using respective small interfering RNAs (siRNAs) (d) in Wnt3a-MCF7 cells and the expression pattern of p68, TCF4 and c-Myc were examined. Densitometry values are given below the blots. **(e)** Expression of p68, TCF4 and c-Myc was determined in EV-MCF7, Wnt3a-MCF7 and p68 knocked down Wnt3a-MCF7 cells by qRT-PCR. **(f)** Wnt3a-MCF7 cells were transfected with GFP-p68 (p68; green) and immunostained with anti-TCF4 antibody (primary) and Alexa Fluor 594 (secondary; red) and visualised under fluorescence microscope. Images were captured at 600X magnification (scale: 10 μm). **(g)** Tcf4 promoter activity was monitored in Wnt3a-MCF7 cells after overexpression of either β-catenin or p68 by transient transfection using luciferase assay.

We have searched the interconnecting effect of these three crucial proteins in terms of their expression levels in serum-starved Wnt3a-MCF7 stable cells. Our results suggest that Wnt3a promotes expression of both p68 and TCF4 proteins and mRNAs (Figures 4a and b), where c-myc, cyclinD1 and axin2 were kept as positive controls. This result indicates the existence of a possible feedback loop. Furthermore, we also examined if overexpression of either β-catenin or p68 in Wnt3a-MCF7 cells had any additive effect on TCF4 expression and observed a significant increase in the level of TCF4 (Figure 4c). To further support the effect of p68-dependent regulation on TCF4, we have knocked down p68 and β-catenin individually in Wnt3a-MCF7 cells and a significant inhibition of Wnt3a-mediated increase of p68, TCF4 and c-Myc was observed (Figures 4d and e). Again, to extend our understanding, we have transiently overexpressed p68 in MCF7 cells to see the effect on TCF4 expression by immunofluorescence. Our results clearly show that p68-transfected cells express more TCF4 (Figure 4f) compared to the untransfected cells. Finally, to confirm this regulation, we have analysed TCF4 promoter activity upon overexpression of either p68 or β-catenin. Here, the induction of TCF4 promoter activity is enhanced by approximately two-fold. This indicates that both molecules are involved in TCF4 gene expression (Figure 4g). Thus, p68 is indeed involved in the regulation of TCF4 and maintains a positive feedback loop within the canonical Wnt signaling pathway.

### Recruitment of β-catenin/TCF4 on p68 promoter is important for its enhanced expression

**Figure 5:**
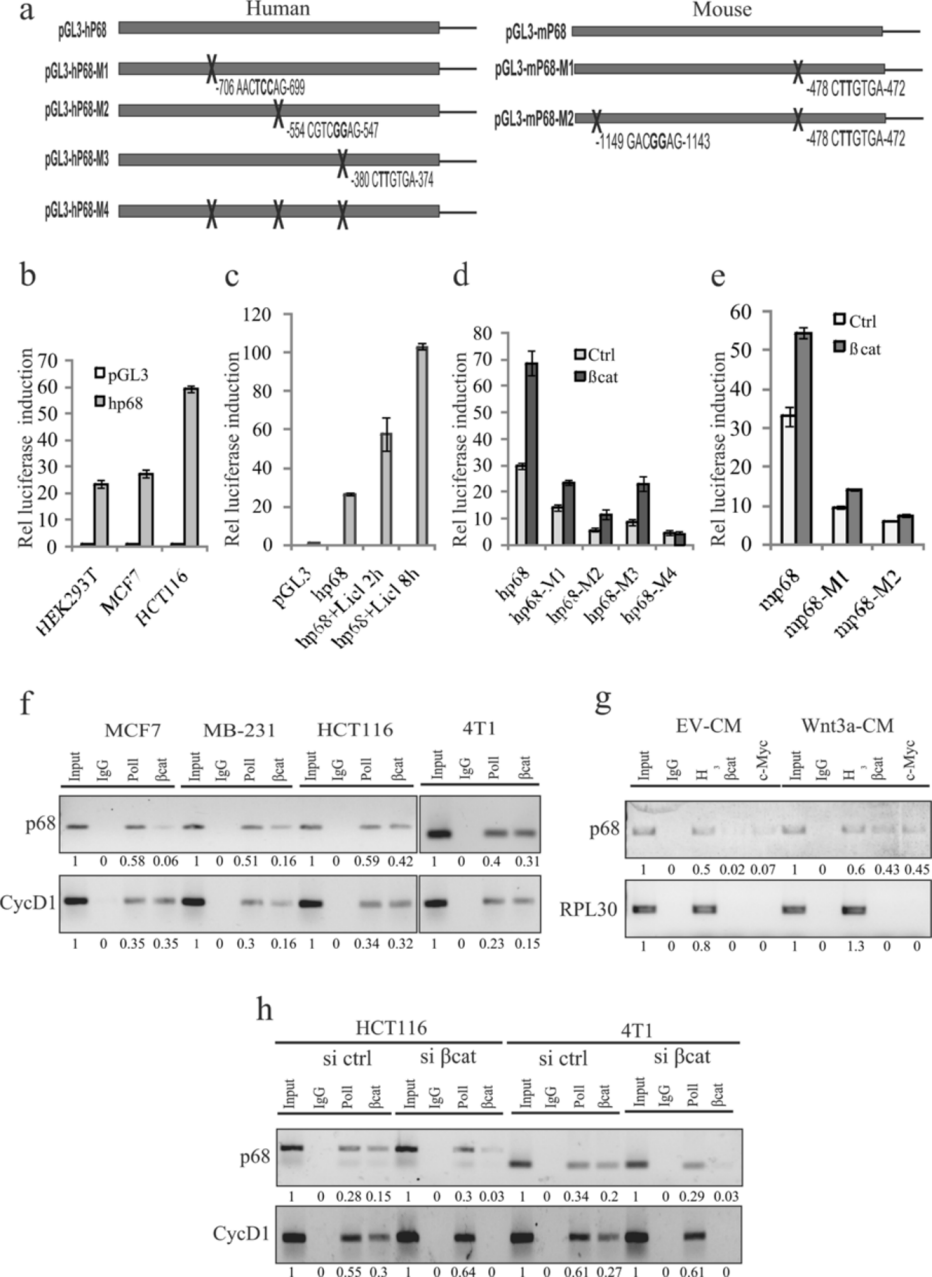
**β-catenin/TCF4 as well as c-Myc regulates p68 promoter. (a)** Schematic representation of human and mouse p68 promoters. **(b)** Activity of human p68 promoter (pGL3-hp68) was determined by luciferase assay in HEK293T, MCF7 and HCT116 cells after 36 h of transfection. Values represent the firefly luciferase activities normalised to renilla luciferase activities. **(c)** Similarly, pGL3-hp68 activity was determined in MCF7 cells treated with 20 mM lithium chloride (LiCl) for 2 h and 8 h. **(d)** Luciferase activities were determined in MCF7 cells transfected with human wild-type pGL3-hp68, or mutated transcription factor 4 (TCF4) constructs like pGL3-hp68-M1, pGL3-hp68-M2, pGL3-hp68-M3 and pGL3-hp68-M4 independently, along with pEGFP-β-catenin. **(e)** Mouse p68 promoter (pGL3-mp68) activities were determined in MCF7 cells transfected with either wild-type pGL3-mp68 or mutated TCF4 sites containing constructs pGL3-mp68-M1 and pGL3-mp68-M2 independently along with pEGFP-β-catenin. **(f)** Cross-linked chromatin fragments of MCF7, MDA-MB 231 and HCT116 (human) as well as 4T1 (mouse) cells were immunoprecipitated with respective antibodies as depicted in the figure. DNAs were isolated and PCR-amplified using primer sets designed from the promoter regions of p68 and cyclin D1 genes. Densitometry values are given below the images. **(g)** MCF7 cells were treated with either Wnt3a-CM or empty vector (EV)-CM (control). DNAs were isolated from cross-linked chromatins immunoprecipitated with the indicated antibodies and PCR-amplified using primer sets designed from the promoter regions of p68 and RPL30 genes. Densitometry values are given below the images. **(h)** β-catenin was knocked down in HCT116 and 4T1 cells using small interfering RNA (siRNA). Control siRNA (si ctrl)-transfected cells were kept as control. DNA fragments were isolated from cross-linked chromatins immunoprecipitated with the indicated antibodies and PCR-amplified using primer sets designed from the promoter regions of p68 and cyclin D1 genes. Input: 2.5% (f and g) or 5% (h) of total DNA isolated from cross-linked chromatin without immunoprecipitation. Densitometry values are given below the images.

It has been well established that nuclear β-catenin is engaged in TCF/LEF protein complexes on its target genes [52]. To further elucidate the molecular mechanism(s) for regulation of p68 gene expression we have cloned and analysed the human and mouse p68 promoter sequences. The human p68 promoter contains three putative TCF4 binding elements corresponding to the core consensus binding sequence (AC/GA/TTCAAAG) at (324-CTTTGGA-317), (554-CGTCAAAG-547) and (706-AACCAAAG-699) and one c-Myc site (380-CACGTGA-374) upstream of the transcription start site. We have mutated two of these three TCF4 sites and the c-Myc site in both human and mouse p68 promoters, represented by schematic diagrams (Figure 5a). The activity of the wild-type p68 promoter was tested in various human cell lines, with differential endogenous β-catenin levels, by luciferase assay. Results indicate that the promoter activity is differentially regulated (Figure 5b), correlating with β-catenin levels (shown in Figure 1a). This regulation is also supported by increased promoter activity due to enhanced β-catenin stabilization in LiCl-treated MCF7 cells (Figure 5c). But all the mutated p68 promoters pGL3-hp68-M1 (putative tcf4 site1), pGL3-hp68-M2 (putative tcf4 site2), pGL3-hp68-M3 (c-myc site) and pGL3-hp68-M4 (all three sites) showed reduced activity, even after β-catenin overexpression, when compared to the wild-type (Figure 5d). The activity of these mutated promoters was found to reduce 2.9-, 5.9-, 3.0- and 16-fold respectively. The mouse p68 promoter consists of one putative tcf4 site (1149-GACAAAG-1143) and one c-Myc site (478-CACGTGA-472; 100% conserved with human sequence) and the activity of the mutated promoters was found to reduce 3.8- and 7.2-fold respectively for c-Myc alone, or when both sites were mutated (Figure 5e).

Next, to examine whether TCF4/β-catenin complex can bind directly to the endogenous p68 promoter, ChIP assay was performed with antibody against β-catenin where RNA polymerase II (Pol II) kept as a positive control. We have found occupancy of β-catenin on the p68 promoter as well as Cyclin D1 promoter (positive control) by ChIP assay in four different cancer cell lines and relatively more in the case of HCT116 and 4T1 (Figure 5f). Similarly, in case of ChIP assay with antibody against TCF4, we found enrichment of p68 as well as c-myc (Figure S6 in Additional file 7). Furthermore, binding of β-catenin and c-Myc was enhanced in Wnt3a-induced cells, keeping RPL30 promoter as the negative control (Figure 5g). Next, β-catenin was knocked down in these cells to investigate the binding of β-catenin to the p68 promoter. It is evident from our results that the occupancy of β-catenin on this promoter depends on the level of β-catenin, thus supporting the involvement of the β-catenin/TCF4 complex in p68 promoter activation (Figure 5h and Figure S6 in Additional file 7). Therefore, it indicates that the β-catenin/TCF4 complex indeed occupies the p68 promoter and the binding is enhanced when β-catenin gets activated. We have also found reduced c-Myc binding in c-myc knockdown cells when compared to the control (Figure S7 in Additional file 8). Altogether, these results highlight that Wnt signaling enhances the β-catenin/TCF4-mediated transcriptional activation of p68 promoter.

### β-catenin/TCF4 mediated upregulation of p68 in breast cancer cells leads to epithelial to mesenchymal transition (EMT)

**Figure 6:**
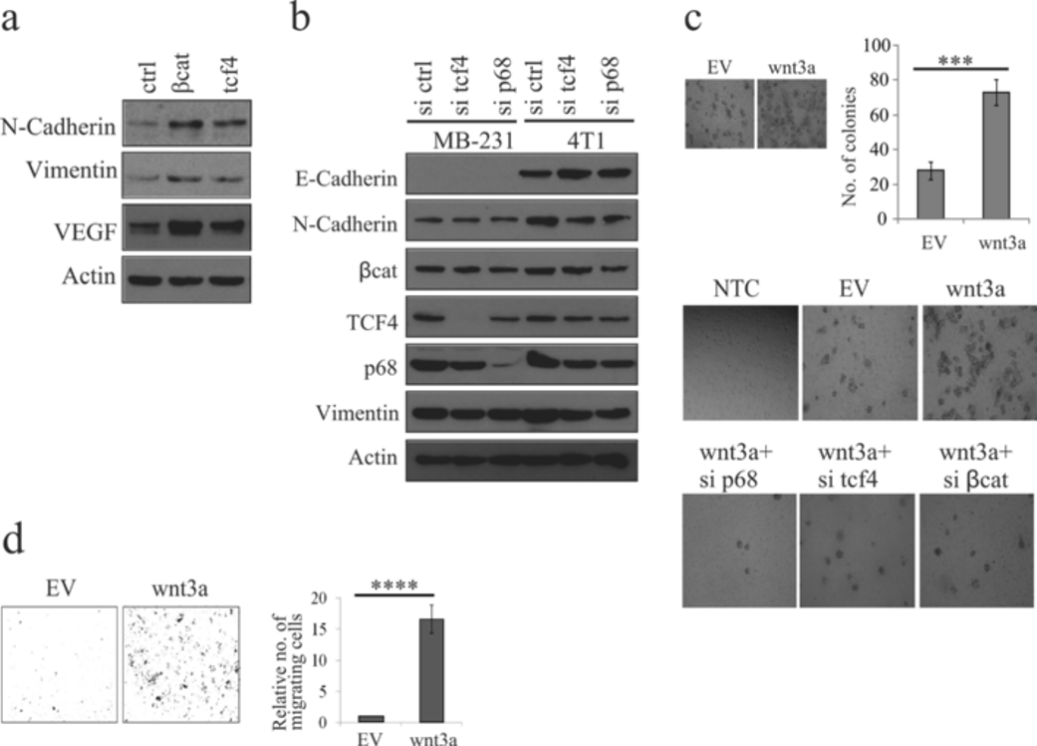
**Wnt signaling-mediated p68 upregulation is important in the induction of EMT. (a)** 4T1 cells were transfected with either β-catenin or transcription factor 4 (TCF4). After 36 h, lysates were prepared and analysed for epithelial to mesenchymal transition (EMT) marker proteins by immunoblotting (IB). **(b)** MDA-MB 231 and 4T1 cells were transfected with scrambled, tcf4 and p68 small interfering RNAs (siRNAs) independently. After 48 h, whole cell lysates (WCLs) were prepared and analysed for EMT marker proteins by IB. **(c)** Wnt3a-MCF7 and empty vector (EV)-MCF7 (control) stable cells were analysed for their anchorage-independent growth by formation of soft-agar colonies. Quantification of the number of colonies and error bars are represented in the figure. Standard deviation (SD) was calculated from three independent biological repeats. Statistical significance was analysed by Student’s *t* test (*P* <0.001) (top). Colony-forming ability of Wnt3a-MCF7 and EV-MCF7 cells were determined after individual knockdown of β-catenin, tcf4 and p68 using respective siRNAs as depicted in the figure (bottom). **(d)** Wnt3a-MCF7 and EV-MCF7 (control) stable cells were analysed for their invasiveness using matrigel invasion assay. Error bars are represented in the figure. SD was calculated from three independent biological repeats. Statistical significance was analysed by Student’s *t* test (*P* <0.001).

EMT is a process by which tumour-associated epithelial cells obtain mesenchymal features resulting in reduced cell-cell contact and increased motility; hence playing a critical role in metastasis. Wnt signaling plays a vital role in EMT [32],[53]. Also, it has been reported that p68 mediates EMT by facilitating nuclear translocation of β-catenin in PDGF-induced cancer cells [32]. To decipher the involvement of β-catenin/TCF4 in regulating p68 and induction of EMT, we have overexpressed either β-catenin or TCF4 in 4T1 cells to enhance the p68 expression. As expected, we have found upregulation of the EMT markers such as N-cadherin, vimentin and VEGF (Figure 6a). Next, respective siRNAs were used to selectively knockdown these genes in MDA-MB 231 and 4T1 cells. Consequent downregulation of EMT-associated markers in either tcf4 or p68 knockdown cells, clearly indicates that increased p68 expression is crucial for Wnt signaling-mediated EMT (Figure 6b).

To assess the anchorage-independent growth and tumourigenic potential of Wnt3a-MCF7 cells, we conducted colony-formation assays. The results indicate that these cells form significantly more number of colonies in comparison to EV-MCF7 cells. Also, individual knockdown of β-catenin, TCF4, or p68 in Wnt3a-MCF7 cells using respective siRNAs showed that knockdown of any of these molecules resulted in reduced number of colonies, indicating that the tumour-promoting ability of β-catenin not only depends on TCF4 but also on p68 (Figure 6c). To further ascertain the role of p68 in invasion of cancer cells, a matrigel invasion assay was performed with Wnt3a-MCF7 cells. These cells showed a significantly higher invasive property compared to control cells (Figure 6d).

### p68 enhances β-catenin/TCF4-dependent breast cancer progression

**Figure 7:**
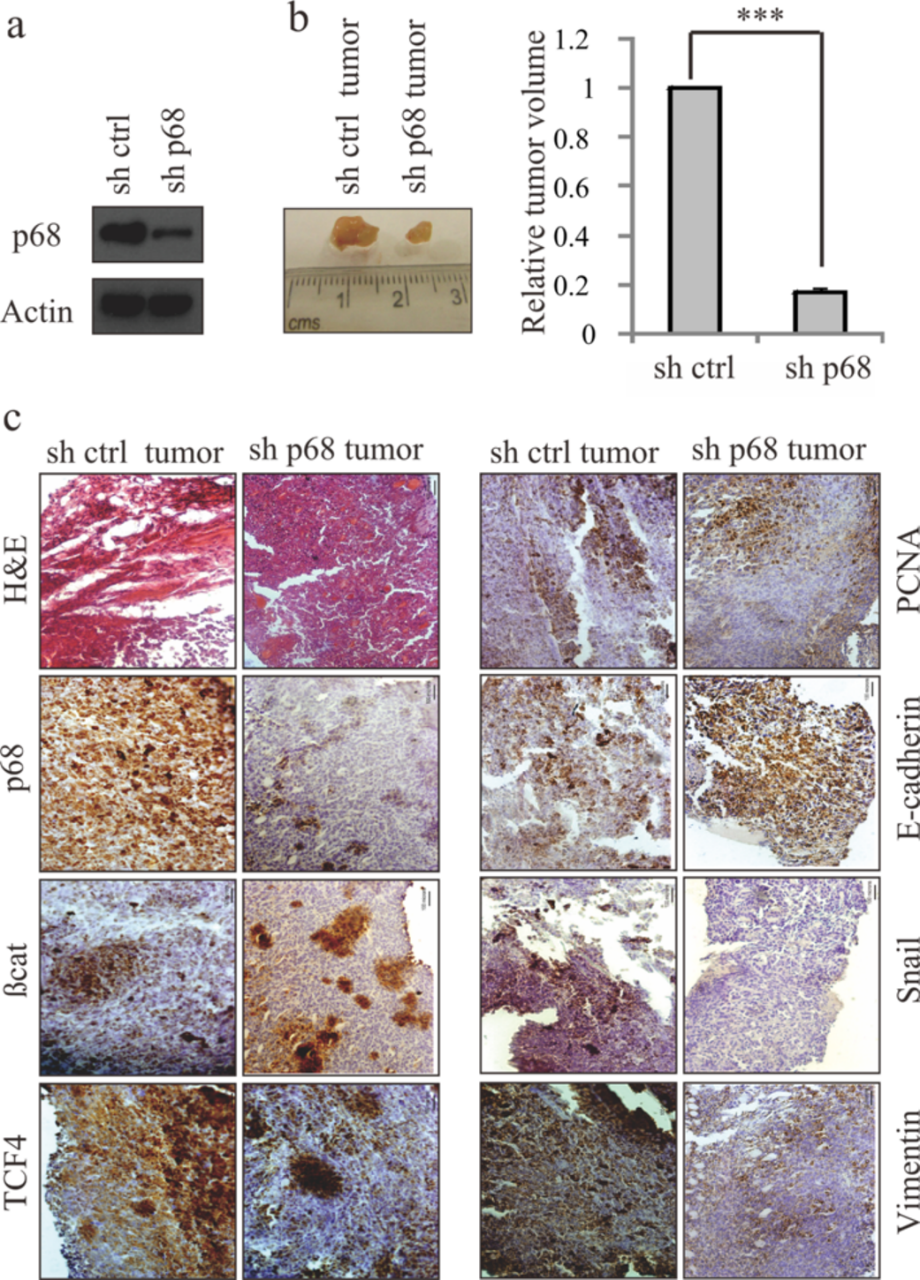
**p68 knockdown reduces breast tumour volume and expression of EMT markers in mice. (a)** p68 expression level was determined in p68 knockdown mouse 4T1 stable cells. Control short hairpin RNA (shRNA)-4T1 stable cells were kept as control. **(b)** Images of mouse breast tumours after 16 days of injections in the mammary fat pad regions with stable cells expressing either control shRNA or p68 shRNA. Tumours obtained from the mammary fat pad regions were analysed for tumour volume (right panel). **(c)** Images of the hematoxylin and eosin (H&E)-stained sections of the tumours and immunohistochemical analysis of the adjacent sections were performed with the indicated antibodies as shown in the figure. Images were captured at magnifications of 200X.

To further confirm that p68 is crucial in Wnt signaling-mediated tumourigenesis, the p68 knockdown and EV stable cells were generated using the mouse syngeneic 4T1 cell line and p68 expression was checked (Figure 7a). These cells were injected into the mouse mammary fat pad to generate tumours. p68 knockdown resulted in reduced tumour volume, indicating the involvement of p68 in tumour progression (Figure 7b). The morphological features of these tumours were ascertained by hematoxylin and eosin (H&E) staining. IHC analysis of the tumour regions confirmed the reduced expression of TCF4, β-catenin and p68 as well as PCNA in the tumours generated with p68 knockdown cells when compared to the tumours generated with EV control cells (Figure 7c). It also exhibited reduced expression of Vimentin and Snail, the EMT markers, and increased expression of E-cadherin, implying involvement of p68 in breast cancer progression and metastasis.

All these results indicate that p68 upregulation through β-catenin/TCF4 signaling is important for enhanced transactivation of β-catenin target genes and thus attributes to the tumourigenic potential of breast cancer cells.

## Discussion

Wnt signaling has been found to be deregulated in most cancers including breast and it plays an important role in tumour progression by upregulating various factors. β-catenin is majorly present in the cell membrane. Wnt3a induces β-catenin stabilisation and promotes its nuclear translocation [54],[55]. Several reports state the importance of p68 overexpression in cancer progression, specifically in breast cancer, which is attributed to gene locus amplification [56]. Here, we have tried to decipher the regulation of p68 gene expression and demonstrated that β-catenin is a critical mediator for p68 expression in cancer cells. A previous study suggested a c-Myc consensus site to be present in the p68 promoter, which is involved in modulating p68 promoter activity [57]-[59]. The current study confirmed that the β-catenin/TCF4 complex regulates p68 promoter, and β-catenin target c-Myc is also involved in this Wnt signaling-mediated p68 gene expression. Our study, using mouse promoter and 4T1 breast cancer cells, indicates that Wnt signaling-mediated p68 gene regulation is conserved in mice. This regulation also exists in human HEK293T and other cancer cells. Thus, β-catenin stabilization and nuclear accumulation through Wnt signaling is vital for upregulation of p68 gene expression. A recent study indicates the presence of putative tcf4 sites in the rat p68 gene and assigned as novel putative target [60]. Here, in this study, we also found the existence of putative tcf4 binding sites in both human and mouse p68 promoters. Our results from promoter analysis and ChIP assay confirm that β-catenin occupies the p68 promoter in both human and mouse cell lines and the occupancy is further increased when β-catenin is either overexpressed or induced by Wnt signaling. Hence, we have emphasized that p68 upregulation by β-catenin/TCF4 may be one of the major contributing factors in breast cancer progression.

Furthermore, we have shown that p68 being the co-activator of β-catenin is also critically involved in the Wnt signaling-mediated expression of TCF4 in breast cancer cells. Thus, p68 appears to govern the critical aspect of β-catenin nuclear function, which is the assembly of a transcription activation complex, by regulating TCF4 expression. Therefore, p68 gene regulation represents an important mechanism for controlling canonical Wnt signaling-mediated proliferation and tumourigenesis.

Nuclear import of β-catenin is another crucial phenomenon in cells in response to Wnt signaling, and it induces transition of epithelial cells to the mesenchymal phenotype and tumour invasion [32],[61]. Our study also established that constitutively overexpressed Wnt3a-MCF7 stable cells showed more tumourigenic potential than the EV-control cells. We have also shown that β-catenin/TCF4-mediated p68 regulation plays an important function in enhanced expression of EMT marker proteins in triple-negative breast cancer cells MDA-MB 231, as well as in mouse 4T1 cells. Again, our knockdown and overexpression studies support the involvement of p68 in the EMT processes, as shown earlier [39]. We further infer that the effect of p68 on the EMT progression is, in part, mediated by its association with β-catenin/TCF4 complex. This is consistent with the fact that many target genes of Wnt/β-catenin signaling have EMT regulatory functions [62]-[64], where p68 may play an important role by inducing β-catenin mediated transcription.

**Figure 8:**
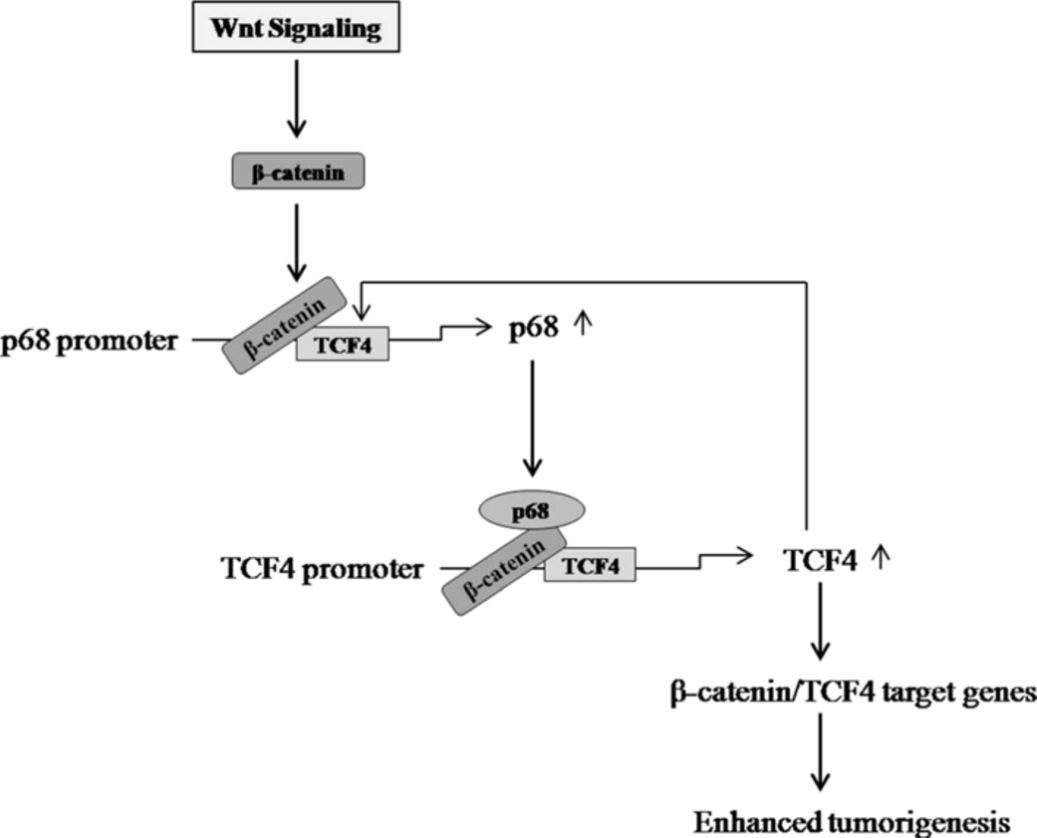
**Schematic representation of β-catenin/TCF4-dependent p68 gene regulation for enhanced tumourigenesis in cancer.** Based on the previous reports and data from the current study, we propose this model. β-catenin can be stabilized and activated by aberrant Wnt signaling that upregulates p68 gene expression, which in turn is involved in increasing transcription factor 4 (TCF4) gene expression and forms a positive feedback loop that ultimately leads to enhanced expression of β-catenin target genes for enhanced tumourigenesis.

Our study further suggests that β-catenin/TCF4 along with c-Myc upregulate p68 in breast cancer cells favouring EMT. Thus, β-catenin/TCF4 and p68 constitute a positive feedback loop essential for β-catenin-mediated processes involved in breast cancer progression. Here, we propose a model (Figure 8) indicating regulation of p68 gene expression in cancer cells by Wnt signaling through a positive feedback mechanism involving β-catenin and TCF4.

## Conclusions

Our findings not only provide an improved understanding of the molecular mechanisms in the context of β-catenin and tumourigenesis but also suggest that p68 may be a crucial target for therapeutic intervention in breast cancer. Although, our data demonstrate that p68 gene regulation by Wnt/β-catenin signaling and its implication in breast cancer, further work is required to understand the p68 upregulation with reference to cancer progression and metastasis.

## Additional files

## Electronic supplementary material

Additional file 1: Table S1.: List of primer sequences used in this study. (DOCX 17 KB)

Additional file 2: Figure S1.: Immunohistochemical analysis of p68, β-catenin and TCF4 in normal human breast tissues samples. **(a)** Immunohistochemistry was performed in normal human breast samples (n = 10) to assess the status of E-cadherin, β-catenin, TCF4 and p68. **(b)** The mean H scores of the desired proteins for each normal breast tissue samples were represented by scatter plot. The images were captured at 100X magnification using BX61 microscope (Olympus). (JPEG 1 MB)

Additional file 3: Figure S2.: Wnt signaling promotes p68 expression. HEK293T, HCT116 and H1299 cells were serum starved for 24 h before treatment with either control condition medium (EV-CM), Wnt3a conditioned medium (Wnt-CM) for another 24 h. Whole cell lysates (WCL) were prepared and analysed by IB to examine the levels of β-catenin and p68. (JPEG 112 KB)

Additional file 4: Figure S3.: GSK3β inactivation regulates p68 expression due to β-catenin stabilization. MCF7 cells were serum starved for 24 h and treated with 20 mM Licl for the indicated periods. Whole cell lysates (WCL) were prepared and analysed by IB to examine the levels of β-catenin, p68 and Cyclin D1. (JPEG 87 KB)

Additional file 5: Figure S4.: TC4 regulates p68 transcript level. HEK293T cells were transfected with either WT-TCF4 or control vector (ctrl). RNAs were isolated from 36 h post-transfected cells and subsequently analysed by qRT-PCR. (JPEG 80 KB)

Additional file 6: Figure S5.: β-Catenin along with c-Myc regulates p68. HEK293T cells were transfected with β-catenin and c-Myc either alone or in combination. WCLs were prepared after 36 h of transfection and analysed by IB to examine the levels of β-catenin, p68 and c-Myc. (JPEG 77 KB)

Additional file 7: Figure S6.: β-catenin/TCF4 complex occupies the p68 promoter. **(a)** Cross-linked chromatins of MCF-7, MDA-MB 231, 4T1, HCT116 cells were immunoprecipitated with anti-TCF4 antibody. **(b)** Cross-linked chromatins of 4T1 and HCT116 cells were transfected with either scrambled siRNA or β-catenin siRNA, and immunoprecipitated with anti-β-catenin antibody. The relative values in both (a) and (b) were normalised to negative control IgG. SEMs were calculated from two independent experiments. (JPEG 2 MB)

Additional file 8: Figure S7.: c-Myc occupies the p68 promoter. Cross-linked chromatin of HCT116 cells transfected with either scrambled or c-Myc siRNA were immunoprecipitated with anti-c-Myc antibody as indicated and subsequently qRT-PCR was performed. The relative values were normalised to IgG (negative control). SEM was calculated from two independent experiments. (JPEG 3 MB)

Below are the links to the authors’ original submitted files for images.

Authors’ original file for figure 1

Authors’ original file for figure 2

Authors’ original file for figure 3

Authors’ original file for figure 4

Authors’ original file for figure 5

Authors’ original file for figure 6

Authors’ original file for figure 7

Authors’ original file for figure 8

Authors’ original file for figure 9

## Abbreviations

AD: actinomycin D
APC: adenomatous polyposis coli
BSA: bovine serum albumin
ChIP: chromatin immunoprecipitation
CHX: cycloheximide
CTNNB1: catenin (cadherin-associated protein) beta 1
DVL1: Dishevelled 1
EMT: epithelial to mesenchymal transition
EV: empty vector
FFPE: formalin-fixed paraffin-embedded
H&E: hematoxylin and eosin
HRP: horseradish peroxidase
IB: immunoblotting
ICC: immunocytochemistry
IgG: immunoglobulin G
IHC: immunohistochemistry
LiCl: lithium chloride
LRP: low-density lipoprotein-related protein
PCNA: proliferating cell nuclear antigen
PDGF: platelet-derived growth factor
SD: standard deviation
sFRPs: secreted Frizzled-related proteins
siRNAs: small interfering RNAs
TCF4: transcription factor 4
WCL: whole cell lysates
WIF-1: Wnt inhibitory factor-1

## References

[1] Brennan KR, Brown AMC (2004). Wnt proteins in mammary development and cancer. J Mammary Gland Biol Neoplasia.

[2] Lin S-Y, Xia W, Wang JC, Kwong KY, Spohn B, Wen Y, Pestell RG (2000). Hung M-C: **β-Catenin, a novel prognostic marker for breast cancer: Its roles in cyclin D1 expression and cancer progression**. Proc Natl Acad Sci U S A.

[3] Prosperi JR, Goss KH (2010). A Wnt-ow of opportunity: targeting the Wnt/beta-catenin pathway in breast cancer. Curr Drug Targets.

[4] Schlosshauer PW, Brown SA, Eisinger K, Yan Q, Guglielminetti ER, Parsons R, Ellenson LH, Kitajewski J (2000). APC truncation and increased β-catenin levels in a human breast cancer cell line. Carcinogenesis.

[5] Zhang J, Li Y, Liu Q, Lu W, Bu G (2010). Wnt signaling activation and mammary gland hyperplasia in MMTV-LRP6 transgenic mice: implication for breast cancer tumorigenesis. Oncogene.

[6] Geyer FC, Lacroix-Triki M, Savage K, Arnedos M, Lambros MB, MacKay A, Natrajan R (2011). Reis-Filho JS: **β-Catenin pathway activation in breast cancer is associated with triple-negative phenotype but not with CTNNB1 mutation**. Mod Pathol.

[7] Jönsson M, Borg A, Nilbert M, Andersson T: Involvement of adenomatous polyposis coli (APC)/beta-catenin signalling in human breast cancer. *Eur J Cancer Oxf Engl 1990* 2000, 36:242–248., 10.1016/s0959-8049(99)00276-2 10741284

[8] Ayyanan A, Civenni G, Ciarloni L, Morel C, Mueller N, Lefort K, Mandinova A, Raffoul W, Fiche M, Dotto GP, Brisken C (2006). Increased Wnt signaling triggers oncogenic conversion of human breast epithelial cells by a Notch-dependent mechanism. Proc Natl Acad Sci U S A.

[9] Benhaj K, Akcali KC, Ozturk M (2006). Redundant expression of canonical Wnt ligands in human breast cancer cell lines. Oncol Rep.

[10] Howe LR, Brown AMC (2004). Wnt signaling and breast cancer. Cancer Biol Ther.

[11] Milovanovic T, Planutis K, Nguyen A, Marsh JL, Lin F, Hope C, Holcombe RF (2004). Expression of Wnt genes and frizzled 1 and 2 receptors in normal breast epithelium and infiltrating breast carcinoma. Int J Oncol.

[12] Yang L, Wu X, Wang Y, Zhang K, Wu J, Yuan Y-C, Deng X, Chen L, Kim CCH, Lau S, Somlo G, Yen Y (2011). FZD7 has a critical role in cell proliferation in triple negative breast cancer. Oncogene.

[13] Liu C-C, Prior J, Piwnica-Worms D, Bu G (2010). LRP6 overexpression defines a class of breast cancer subtype and is a target for therapy. Proc Natl Acad Sci.

[14] Nagahata T, Shimada T, Harada A, Nagai H, Onda M, Yokoyama S, Shiba T, Jin E, Kawanami O, Emi M (2003). Amplification, up-regulation and over-expression of DVL-1, the human counterpart of the Drosophila disheveled gene, in primary breast cancers. Cancer Sci.

[15] Klarmann GJ, Decker A, Farrar WL (2008). Epigenetic gene silencing in the Wnt pathway in breast cancer. Epigenetics.

[16] Suzuki H, Toyota M, Caraway H, Gabrielson E, Ohmura T, Fujikane T, Nishikawa N, Sogabe Y, Nojima M, Sonoda T, Mori M, Hirata K, Imai K, Shinomura Y, Baylin SB, Tokino T (2008). Frequent epigenetic inactivation of Wnt antagonist genes in breast cancer. Br J Cancer.

[17] Veeck J, Niederacher D, An H, Klopocki E, Wiesmann F, Betz B, Galm O, Camara O, Dürst M, Kristiansen G, Huszka C, Knüchel R, Dahl E (2006). Aberrant methylation of the Wnt antagonist SFRP1 in breast cancer is associated with unfavourable prognosis. Oncogene.

[18] Nakopoulou L, Mylona E, Papadaki I, Kavantzas N, Giannopoulou I, Markaki S, Keramopoulos A (2006). Study of phospho-β-catenin subcellular distribution in invasive breast carcinomas in relation to their phenotype and the clinical outcome. Mod Pathol.

[19] Lane DP, Hoeffler WK (1980). SV40 large T shares an antigenic determinant with a cellular protein of molecular weight 68,000. Nature.

[20] Ford MJ, Anton IA, Lane DP (1988). Nuclear protein with sequence homology to translation initiation factor eIF-4A. Nature.

[21] Fukuda T, Yamagata K, Fujiyama S, Matsumoto T, Koshida I, Yoshimura K, Mihara M, Naitou M, Endoh H, Nakamura T, Akimoto C, Yamamoto Y, Katagiri T, Foulds C, Takezawa S, Kitagawa H, Takeyama K, O’Malley BW, Kato S (2007). DEAD-box RNA helicase subunits of the Drosha complex are required for processing of rRNA and a subset of microRNAs. Nat Cell Biol.

[22] Abdelhaleem M (2005). RNA helicases: regulators of differentiation. Clin Biochem.

[23] Fuller-Pace FV (2006). DExD/H box RNA helicases: multifunctional proteins with important roles in transcriptional regulation. Nucleic Acids Res.

[24] Rossler OG, Straka A, Stahl H (2001). Rearrangement of structured RNA via branch migration structures catalysed by the highly related DEAD-box proteins p68 and p72. Nucleic Acids Res.

[25] Métivier R, Penot G, Hübner MR, Reid G, Brand H, Kos M, Gannon F (2003). Estrogen receptor-α directs ordered, cyclical, and combinatorial recruitment of cofactors on a natural target promoter. Cell.

[26] Watanabe M, Yanagisawa J, Kitagawa H, Takeyama K, Ogawa S, Arao Y, Suzawa M, Kobayashi Y, Yano T, Yoshikawa H, Masuhiro Y, Kato S (2001). A subfamily of RNA-binding DEAD-box proteins acts as an estrogen receptor α coactivator through the N-terminal activation domain (AF-1) with an RNA coactivator, SRA. EMBO J.

[27] Clark EL, Coulson A, Dalgliesh C, Rajan P, Nicol SM, Fleming S, Heer R, Gaughan L, Leung HY, Elliott DJ, Fuller-Pace FV, Robson CN (2008). The RNA helicase p68 is a novel androgen receptor coactivator involved in splicing and is overexpressed in prostate cancer. Cancer Res.

[28] Bates GJ, Nicol SM, Wilson BJ, Jacobs A-MF, Bourdon J-C, Wardrop J, Gregory DJ, Lane DP, Perkins ND, Fuller-Pace FV (2005). The DEAD box protein p68: a novel transcriptional coactivator of the p53 tumour suppressor. EMBO J.

[29] Caretti G, Schiltz RL, Dilworth FJ, Padova MD, Zhao P, Ogryzko V, Fuller-Pace FV, Hoffman EP, Tapscott SJ, Sartorelli V (2006). The RNA helicases p68/p72 and the noncoding RNA SRA are coregulators of MyoD and skeletal muscle differentiation. Dev Cell.

[30] Shin S, Rossow KL, Grande JP, Janknecht R (2007). Involvement of RNA Helicases p68 and p72 in Colon Cancer. Cancer Res.

[31] Yang L, Lin C, Liu Z-R (2006). P68 RNA helicase mediates PDGF-induced epithelial mesenchymal transition by displacing axin from β-catenin. Cell.

[32] Yang L, Lin C, Zhao S, Wang H, Liu Z-R (2007). Phosphorylation of p68 RNA helicase plays a role in platelet-derived growth factor-induced cell proliferation by up-regulating cyclin D1 and c-Myc expression. J Biol Chem.

[33] Jacobs A-M, Nicol SM, Hislop RG, Jaffray EG, Hay RT, Fuller-Pace FV (2007). SUMO modification of the DEAD box protein p68 modulates its transcriptional activity and promotes its interaction with HDAC1. Oncogene.

[34] Causevic M, Hislop RG, Kernohan NM, Carey FA, Kay RA, Steele RJ, Fuller-Pace FV (2001). Overexpression and poly-ubiquitylation of the DEAD-box RNA helicase p68 in colorectal tumours. 20. Oncogene.

[35] Mooney SM, Grande JP, Salisbury JL, Janknecht R (2010). Sumoylation of p68 and p72 RNA helicases affects protein stability and transactivation potential. Biochemistry (Mosc).

[36] Beier UH, Maune S, Meyer JE, Görögh T (2006). Overexpression of p68 mRNA in head and neck squamous cell carcinoma cells. Anticancer Res.

[37] Stevenson RJ, Hamilton SJ, MacCallum DE, Hall PA, Fuller-Pace FV (1998). Expression of the “DEAD box” RNA helicase p68 is developmentally and growth regulated and correlates with organ differentiation/maturation in the fetus. J Pathol.

[38] Bhattacharya S, Ghosh MK (2014). HAUSP, a novel deubiquitinase for Rb - MDM2 the critical regulator. FEBS J.

[39] Carter CL, Lin C, Liu C-Y, Yang L, Liu Z-R (2010). Phosphorylated p68 RNA helicase activates Snail1 transcription by promoting HDAC1 dissociation from the Snail1 promoter. Oncogene.

[40] Paul I, Ahmed SF, Bhowmik A, Deb S, Ghosh MK (2013). The ubiquitin ligase CHIP regulates c-Myc stability and transcriptional activity. Oncogene.

[41] Guturi KKN, Mandal T, Chatterjee A, Sarkar M, Bhattacharya S, Chatterjee U, Ghosh MK (2012). Mechanism of β-catenin-mediated transcriptional regulation of epidermal growth factor receptor expression in glycogen synthase kinase 3 β-inactivated prostate cancer cells. J Biol Chem.

[42] Sha J, Ghosh MK, Zhang K, Harter ML (2010). E1A interacts with Two opposing transcriptional pathways to induce quiescent cells into S phase. J Virol.

[43] Bhowmik A, Das N, Pal U, Mandal M, Bhattacharya S, Sarkar M, Jaisankar P, Maiti NC, Ghosh MK (2013). 2,2’-diphenyl-3,3’-diindolylmethane: a potent compound induces apoptosis in breast cancer cells by inhibiting EGFR pathway. PLoS One.

[44] Mandal T, Bhowmik A, Chatterjee A, Chatterjee U, Chatterjee S, Ghosh MK (2014). Reduced phosphorylation of Stat3 at Ser-727 mediated by casein kinase 2 - Protein phosphatase 2A enhances Stat3 Tyr-705 induced tumorigenic potential of glioma cells. Cell Signal.

[45] Chatterjee A, Chatterjee U, Ghosh MK (2013). Activation of protein kinase CK2 attenuates FOXO3a functioning in a PML-dependent manner: implications in human prostate cancer. Cell Death Dis.

[46] GraphPad QuickCalcs. [http://www.graphpad.com/quickcalcs/index.cfm]

[47] Yang L, Lin C, Liu Z-R (2005). Phosphorylations of DEAD box p68 RNA helicase are associated with cancer development and cell proliferation. Mol Cancer Res.

[48] Mastracci TL, Tjan S, Bane AL, O’Malley FP, Andrulis IL (2005). E-cadherin alterations in atypical lobular hyperplasia and lobular carcinoma in situ of the breast. Mod Pathol.

[49] Wang D, Huang J, Hu Z (2012). RNA helicase DDX5 regulates microRNA expression and contributes to cytoskeletal reorganization in basal breast cancer cells. Mol Cell Proteomics.

[50] Yang S-Z, Kohno N, Yokoyama A, Kondo K, Hamada H, Hiwada K (2001). Decreased E-cadherin augments β-catenin nuclear localization: Studies in breast cancer cell lines. Int J Oncol.

[51] Saegusa M, Hashimura M, Kuwata T, Hamano M, Okayasu I (2005). Upregulation of TCF4 expression as a transcriptional target of β-catenin/p300 complexes during trans-differentiation of endometrial carcinoma cells. Lab Invest.

[52] Mosimann C, Hausmann G, Basler K (2009). Beta-catenin hits chromatin: regulation of Wnt target gene activation. Nat Rev Mol Cell Biol.

[53] Kahlina K, Goren I, Pfeilschifter J, Frank S (2004). p68 DEAD Box RNA helicase expression in keratinocytes regulation, nucleolar localization, and functional connection to proliferation and vascular endothelial growth factor gene expression. J Biol Chem.

[54] Liu J, Ding X, Tang J, Cao Y, Hu P, Zhou F, Shan X, Cai X, Chen Q, Ling N, Zhang B, Bi Y, Chen K, Ren H, Huang A, He T-C, Tang N (2011). Enhancement of canonical Wnt/β-catenin signaling activity by HCV core protein promotes cell growth of hepatocellular carcinoma cells. PLoS One.

[55] Paul I, Bhattacharya S, Chatterjee A, Ghosh MK (2013). Current understanding on EGFR and Wnt/β-catenin signaling in glioma and their possible crosstalk. Genes Cancer.

[56] Mazurek A, Luo W, Krasnitz A, Hicks J, Powers RS, Stillman B (2012). DDX5 regulates DNA replication and is required for cell proliferation in a subset of breast cancer cells. Cancer Discov.

[57] Li Z, Calcar SV, Qu C, Cavenee WK, Zhang MQ, Ren B (2003). A global transcriptional regulatory role for c-Myc in Burkitt’s lymphoma cells. Proc Natl Acad Sci.

[58] Menssen A, Hermeking H (2002). Characterization of the c-MYC-regulated transcriptome by SAGE: identification and analysis of c-MYC target genes. Proc Natl Acad Sci.

[59] Rössler OG, Hloch P, Schütz N, Weitzenegger T, Stahl H (2000). Structure and expression of the human p68 RNA helicase gene. Nucleic Acids Res.

[60] Nelson WJ, Nusse R (2004). Convergence of Wnt, ß-catenin, and cadherin pathways. Science.

[61] Schmalhofer O, Brabletz S, Brabletz T (2009). E-cadherin, beta-catenin, and ZEB1 in malignant progression of cancer. Cancer Metastasis Rev.

[62] Crawford HC, Fingleton BM, Rudolph-Owen LA, Goss KJ, Rubinfeld B, Polakis P, Matrisian LM (1999). The metalloproteinase matrilysin is a target of beta-catenin transactivation in intestinal tumors. Oncogene.

[63] Easwaran V, Lee SH, Inge L, Guo L, Goldbeck C, Garrett E, Wiesmann M, Garcia PD, Fuller JH, Chan V, Randazzo F, Gundel R, Warren RS, Escobedo J, Aukerman SL, Taylor RN (2003). Fantl WJ: **beta-Catenin regulates vascular endothelial growth factor expression in colon cancer**. Cancer Res.

[64] Sánchez-Tilló E, de Barrios O, Siles L, Cuatrecasas M, Castells A (2011). Postigo A: **β-catenin/TCF4 complex induces the epithelial-to-mesenchymal transition (EMT)-activator ZEB1 to regulate tumor invasiveness**. Proc Natl Acad Sci U S A.

