# Supplementary material for: DEAD-box protein p68 is regulated by β-catenin/transcription factor 4 to maintain a positive feedback loop in control of breast cancer progression
Source: Breast Cancer Res. 2014 Dec 12;16:496. doi: 10.1186/s13058-014-0496-5 (PMC4308923; doi:10.1186/s13058-014-0496-5)
Supplement: Supplementary file 1 — Additional file 1: Table S1.: List of primer sequences used in this study. (DOCX 17 KB) [file 13058_2014_496_MOESM1_ESM.docx]

**Table S1. List of primer sequences used in this study.**

| 1. ***Primer Sequences used for Cloning and Sub-Cloning*-** | |
| --- | --- |
| mp68-Prom- F | 5-AATGGTACCAGATACCCCACAAAAATTCGG-3 |
| mp68-Prom- R | 5-AATAGATCTGGCGTCAATGGTGGCGG-3 |
| p68-Prom- F | 5-ATGGGTACCCGAAGAGGTGGTGAAGCTGAGAG-3 |
| p68-Prom- R | 5-TGGAGATCTCGCGGTCTCGGTCACTCGAATAACC-3 |
| βcat-GFP- F | 5-AATGGTACCATGGCTACTCAAGCTGACCTG-3 |
| βcat-GFP- R | 5-AATGGATCCTTACAGGTCAGTATCAAACC-3 |
| wnt3a-myc-his- F | 5-AATCTCGAGGCGATGGCCCCACTCGGATACTTC-3 |
| wnt3a-myc-his- R | 5-AATGAATTCCTACTTGCAGGTGTGCACGTCGTAG-3 |
| 1. ***Primer Sequences used for Mutagenesis*-** | |
| mp68-Prom M2- F | 5- GTCCCAGGCTCGACGGAGAGTTCCTATC-3 |
| mp68-Prom M2- R | 5-GATAGGAACTCTCCGTCGAGCCTGGGAC-3 |
| mp68-Prom M1- F | 5-CCTGGGTCCGATCTTGTGATGAGGCCAAGC-3 |
| mp68-Prom M1- R | 5-GCTTGGCCTCATCACAAGATCGGACCCAGG-3 |
| p68-Prom M2- F | 5-CGACCAAAACCCGTCGGAGGATTATCAGAC-3 |
| p68-Prom M2- R | 5-GTCTGATAATCCTCCGACGGGTTTTGGTCG-3 |
| p68-Prom M3- F | 5-GTGGTACAGCTTTGTGACAACGCCAGG-3 |
| p68-Prom M3- R | 5-TGGCGTTGTCACAAAGCTGTACCACACC-3 |
| p68-Prom M1- F | 5-ACGCACGAACTCCAGCGCGGGGAA-3 |
| p68-Prom M1- R | 5-CCGCGCTGGAGTTCGTGCGTCCG-3 |
| 1. ***Primer Sequences used for qRT-PCR*-** | |
| RT-mp68- F | 5-CGGGATCGAGGGTTTGGTG-3 |
| RT-mp68- R | 5-GCAGCTCATCAAGATTCCACTTC-3 |
| RT-mβcat- F | 5-ATGGAGCCGGACAGAAAAGC-3 |
| RT-mβcat- R | 5-CTTGCCACTCAGGGAAGGA-3 |
| RT-cyc D1- F | 5-CCGTCCATGCGGAAGATC-3 |
| RT-cyc D1- R | 5-GAAGACCTCCTCCTCGCACT-3 |
| RT-p68- F | 5-AGAGGTTCAGGTCGTTCCAGG-3 |
| RT-p68- R | 5-GGAATATCCTGTTGGCATTGG-3 |
| RT-cmyc- F | 5-CCAACAGGAACTATGACCTCGACTAC-3 |
| RT-cmyc- R | 5-CTCGAATTTCTTCCAGATATCCT-3 |
| RT-βcat- F | 5-TACCTCCCAAGTCCTGTATGAG-3 |
| RT-βcat- R | 5-TGAGCAGCATCAAACTGTGTAG-3 |
| RT-axin2- F | 5-CAAGGGCCAGGTCACCAA-3 |
| RT-axin2- R | 5-CCCCCAACCCATCTTCGT-3 |
| 1. ***Primer Sequences used for qRT-PCR and PCR in the ChIP Experiment*-** | |
| c-myc-ChIP- F | 5-AGTTAACGGTTTTTTCACAAGG-3 |
| c-myc-ChIP- R | 5-ATAACCCAGCAACGCATTGC-3 |
| p68-ChIP- F | 5-ACGCACGAACCAAAGCGCGGGG-3 |
| p68-ChIP- R | 5-TGGCGTTGTCACGTGGCTGTAC-3 |
| mp68-ChIP- F | 5-AATGGTACCAGATACCCCAC-3 |
| mP68-ChIP- R | 5-GATAGGAACTCTTTGTCGAGCCT-3 |

**Note:** Prom, promoter; m, mouse; M, mutagenesis; RT, reverse transcription; F, forward and R, reverse.
