# Supplementary figures and images for: DEAD-box protein p68 is regulated by β-catenin/transcription factor 4 to maintain a positive feedback loop in control of breast cancer progression
Source: Breast Cancer Res. 2014 Dec 12;16:496. doi: 10.1186/s13058-014-0496-5 (PMC4308923; doi:10.1186/s13058-014-0496-5)

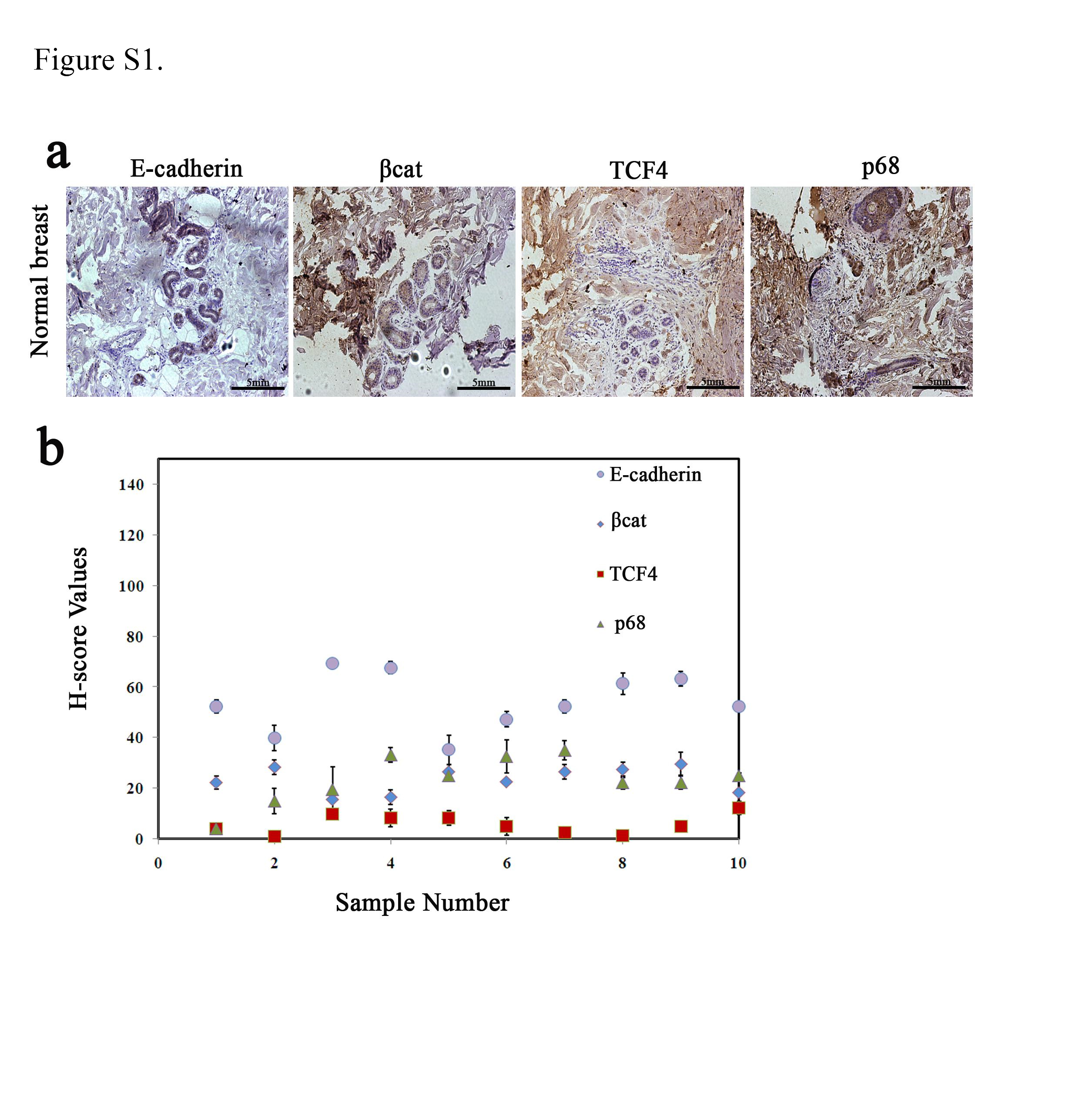

Supplement: Supplementary file 2 — Additional file 2: Figure S1.: Immunohistochemical analysis of p68, β-catenin and TCF4 in normal human breast tissues samples. (a) Immunohistochemistry was performed in normal human breast samples (n = 10) to assess the status of E-cadherin, β-catenin, TCF4 and p68. (b) The mean H scores of the desired proteins for each normal breast tissue samples were represented by scatter plot. The images were captured at 100X magnification using BX61 microscope (Olympus). (JPEG 1 MB) [file 13058_2014_496_MOESM2_ESM.jpeg]

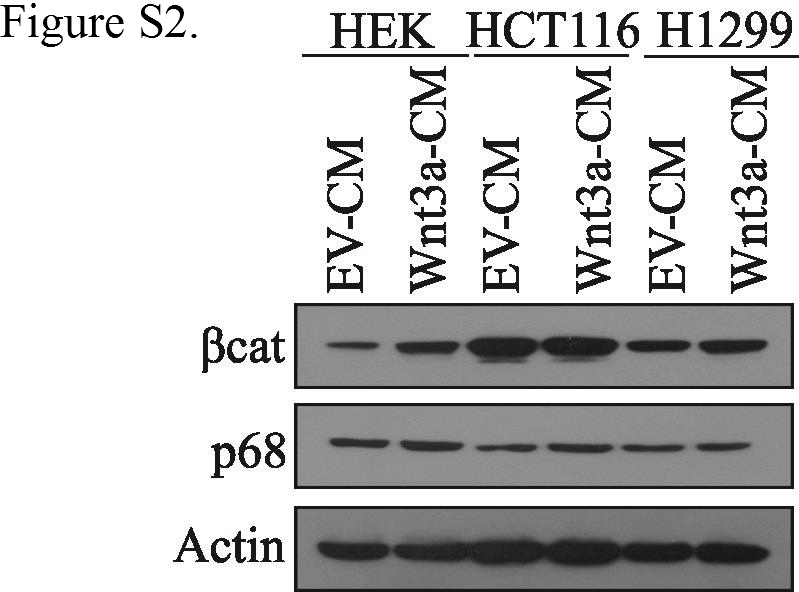

Supplement: Supplementary file 3 — Additional file 3: Figure S2.: Wnt signaling promotes p68 expression. HEK293T, HCT116 and H1299 cells were serum starved for 24 h before treatment with either control condition medium (EV-CM), Wnt3a conditioned medium (Wnt-CM) for another 24 h. Whole cell lysates (WCL) were prepared and analysed by IB to examine the levels of β-catenin and p68. (JPEG 112 KB) [file 13058_2014_496_MOESM3_ESM.jpeg]

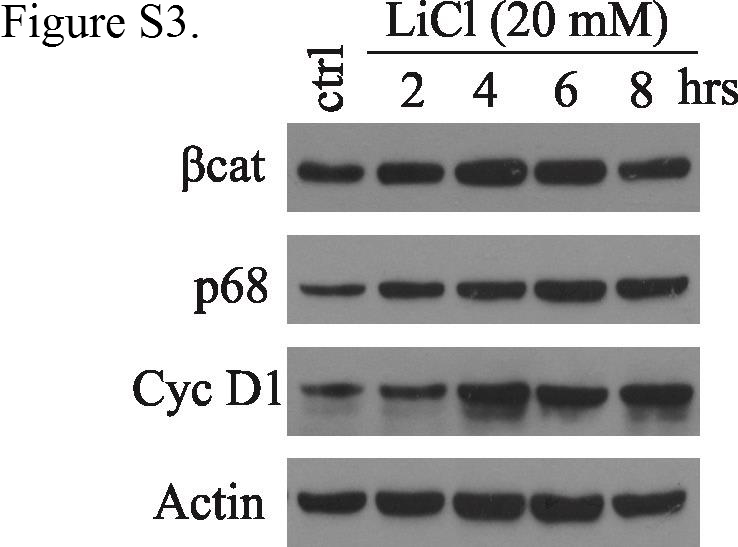

Supplement: Supplementary file 4 — Additional file 4: Figure S3.: GSK3β inactivation regulates p68 expression due to β-catenin stabilization. MCF7 cells were serum starved for 24 h and treated with 20 mM Licl for the indicated periods. Whole cell lysates (WCL) were prepared and analysed by IB to examine the levels of β-catenin, p68 and Cyclin D1. (JPEG 87 KB) [file 13058_2014_496_MOESM4_ESM.jpeg]

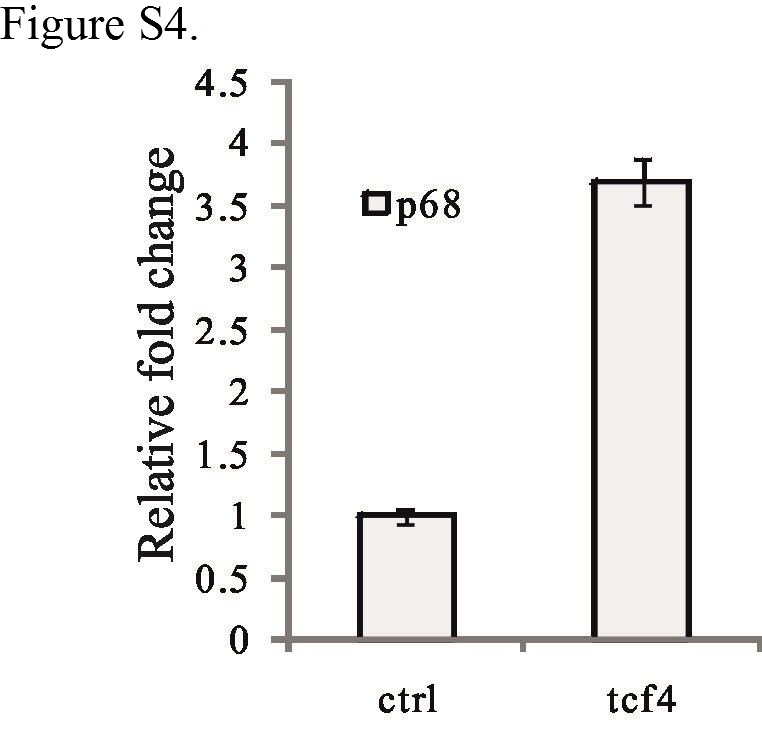

Supplement: Supplementary file 5 — Additional file 5: Figure S4.: TC4 regulates p68 transcript level. HEK293T cells were transfected with either WT-TCF4 or control vector (ctrl). RNAs were isolated from 36 h post-transfected cells and subsequently analysed by qRT-PCR. (JPEG 80 KB) [file 13058_2014_496_MOESM5_ESM.jpeg]

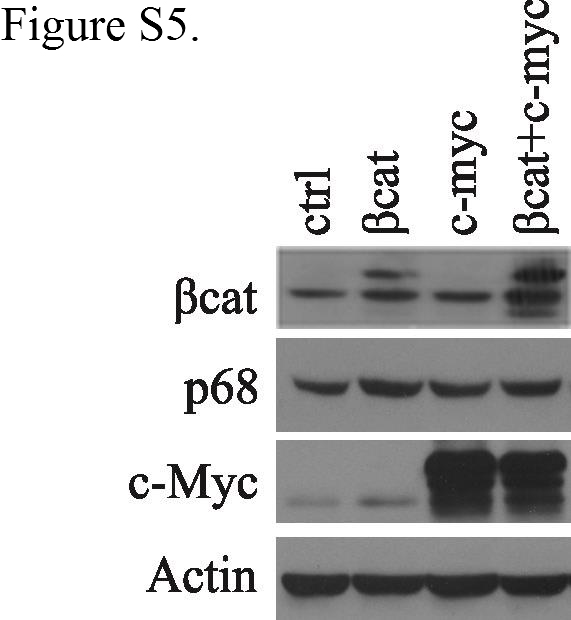

Supplement: Supplementary file 6 — Additional file 6: Figure S5.: β-Catenin along with c-Myc regulates p68. HEK293T cells were transfected with β-catenin and c-Myc either alone or in combination. WCLs were prepared after 36 h of transfection and analysed by IB to examine the levels of β-catenin, p68 and c-Myc. (JPEG 77 KB) [file 13058_2014_496_MOESM6_ESM.jpeg]

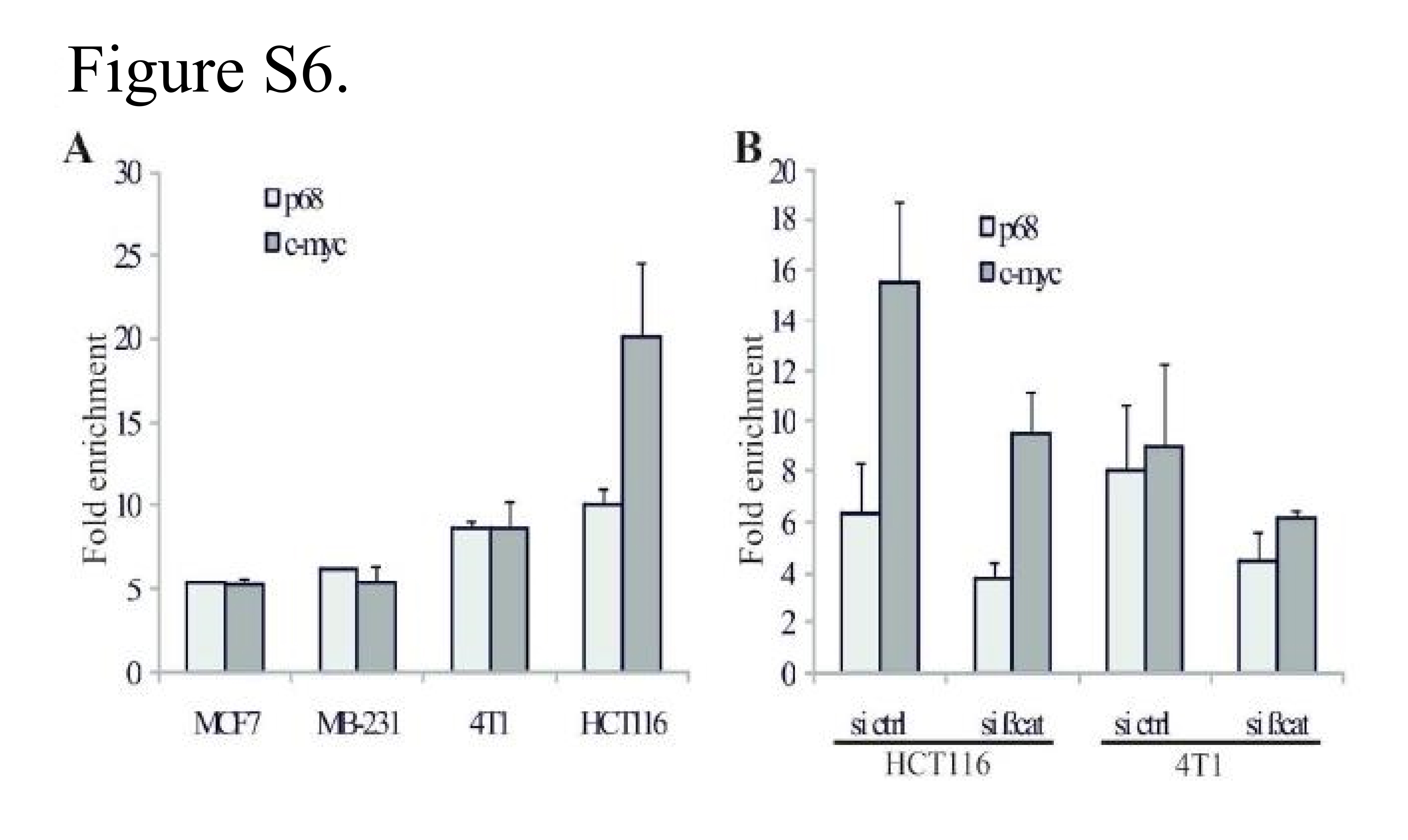

Supplement: Supplementary file 7 — Additional file 7: Figure S6.: β-catenin/TCF4 complex occupies the p68 promoter. (a) Cross-linked chromatins of MCF-7, MDA-MB 231, 4T1, HCT116 cells were immunoprecipitated with anti-TCF4 antibody. (b) Cross-linked chromatins of 4T1 and HCT116 cells were transfected with either scrambled siRNA or β-catenin siRNA, and immunoprecipitated with anti-β-catenin antibody. The relative values in both (a) and (b) were normalised to negative control IgG. SEMs were calculated from two independent experiments. (JPEG 2 MB) [file 13058_2014_496_MOESM7_ESM.jpeg]

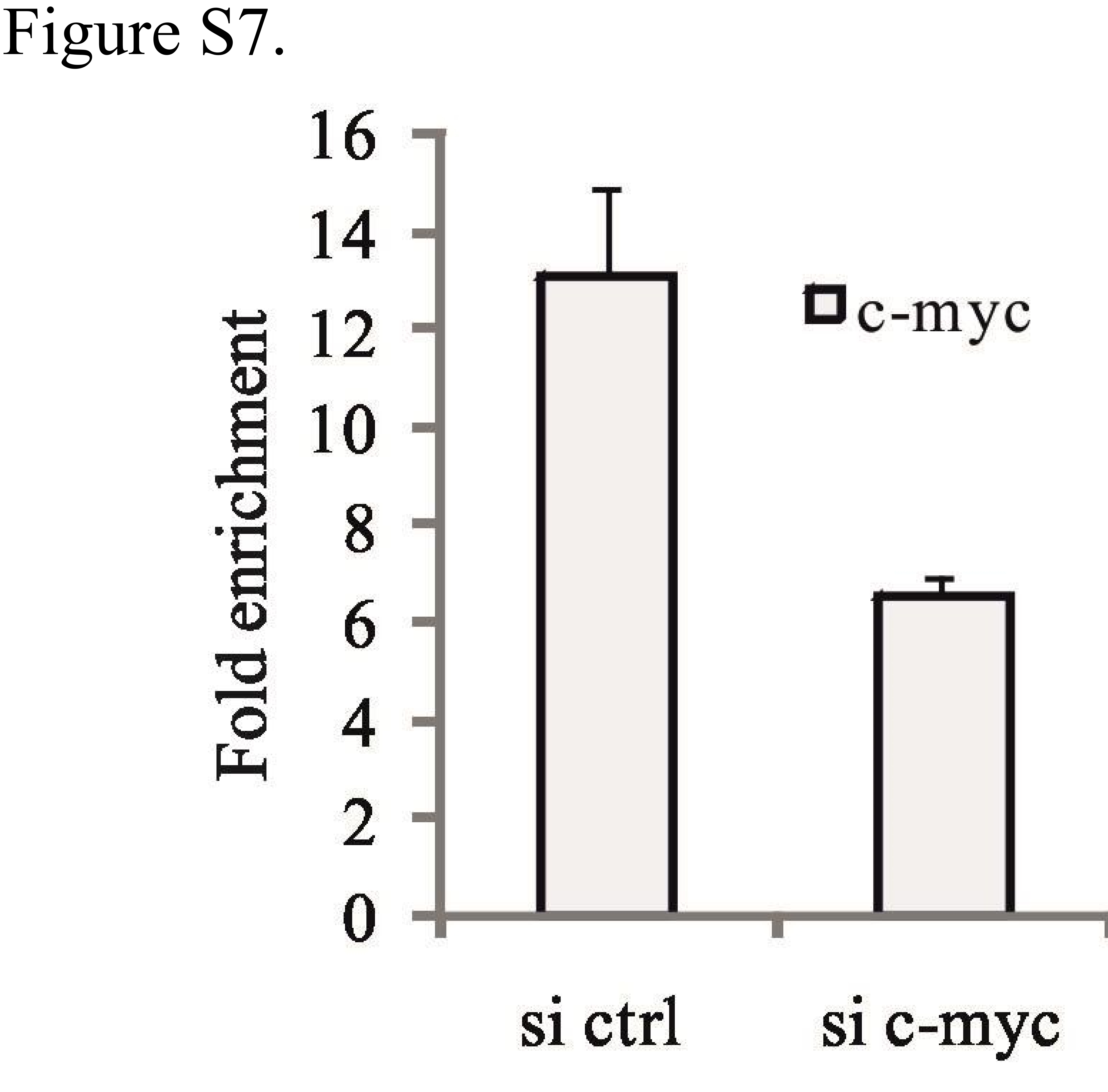

Supplement: Supplementary file 8 — Additional file 8: Figure S7.: c-Myc occupies the p68 promoter. Cross-linked chromatin of HCT116 cells transfected with either scrambled or c-Myc siRNA were immunoprecipitated with anti-c-Myc antibody as indicated and subsequently qRT-PCR was performed. The relative values were normalised to IgG (negative control). SEM was calculated from two independent experiments. (JPEG 3 MB) [file 13058_2014_496_MOESM8_ESM.jpeg]

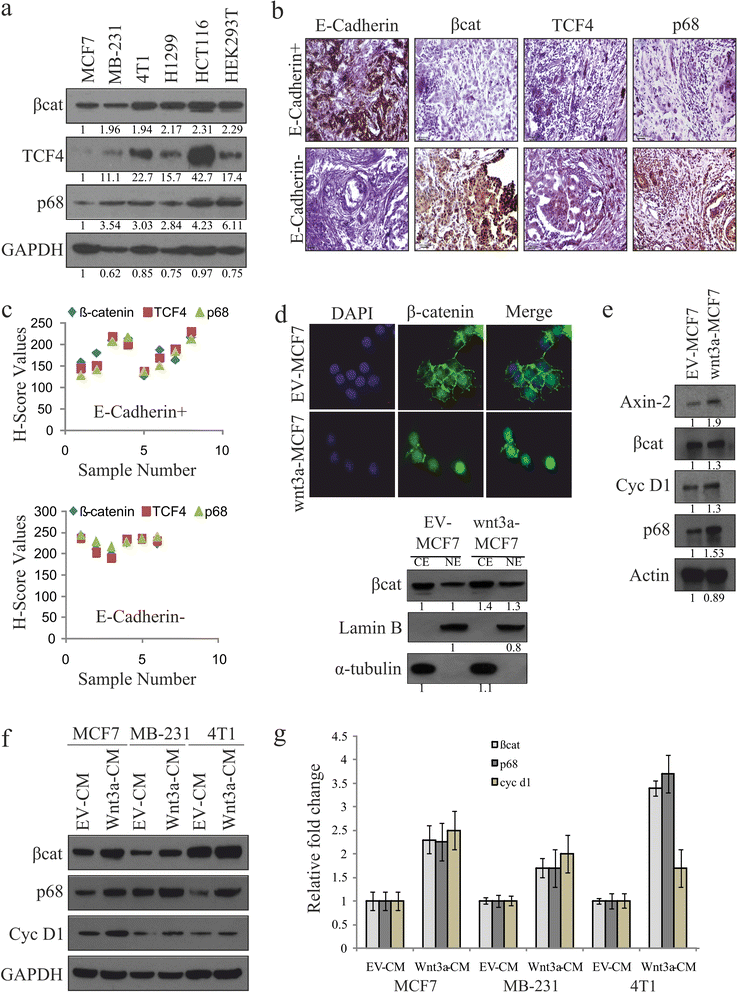

Supplement: Supplementary file 9 — Authors’ original file for figure 1 [file 13058_2014_496_MOESM9_ESM.gif]

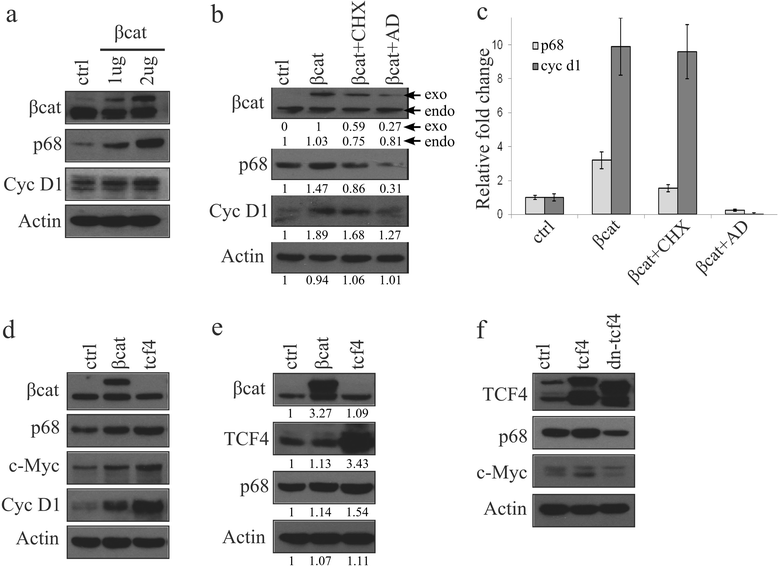

Supplement: Supplementary file 10 — Authors’ original file for figure 2 [file 13058_2014_496_MOESM10_ESM.gif]

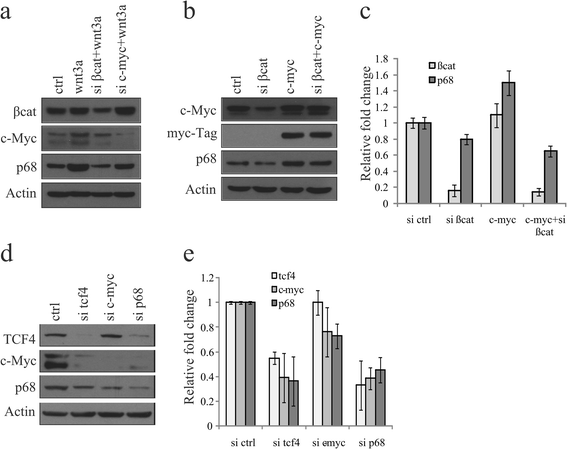

Supplement: Supplementary file 11 — Authors’ original file for figure 3 [file 13058_2014_496_MOESM11_ESM.gif]

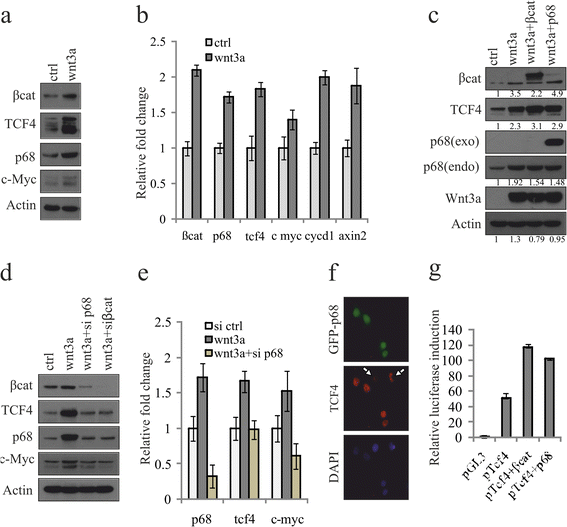

Supplement: Supplementary file 12 — Authors’ original file for figure 4 [file 13058_2014_496_MOESM12_ESM.gif]

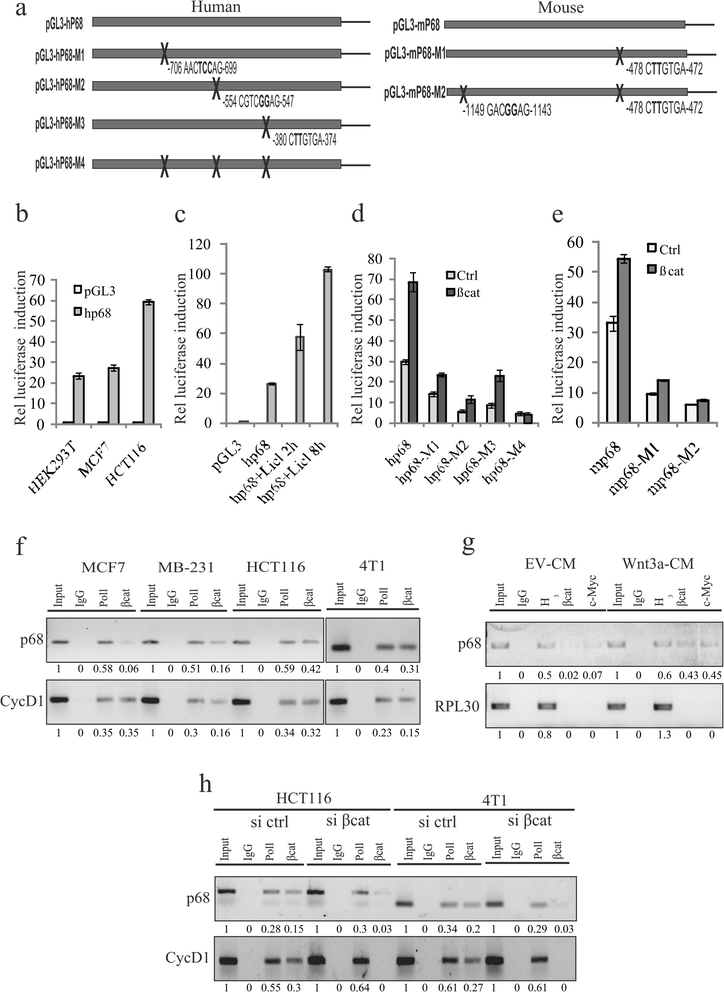

Supplement: Supplementary file 13 — Authors’ original file for figure 5 [file 13058_2014_496_MOESM13_ESM.gif]

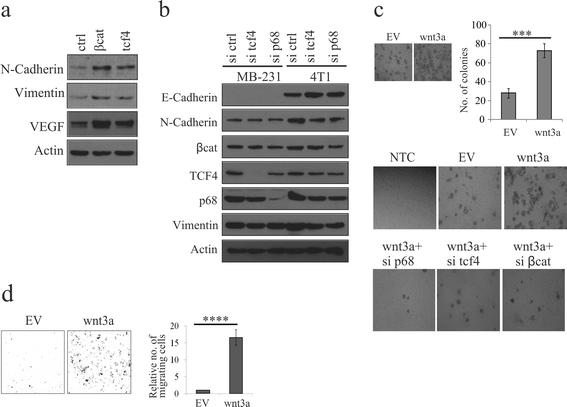

Supplement: Supplementary file 14 — Authors’ original file for figure 6 [file 13058_2014_496_MOESM14_ESM.gif]

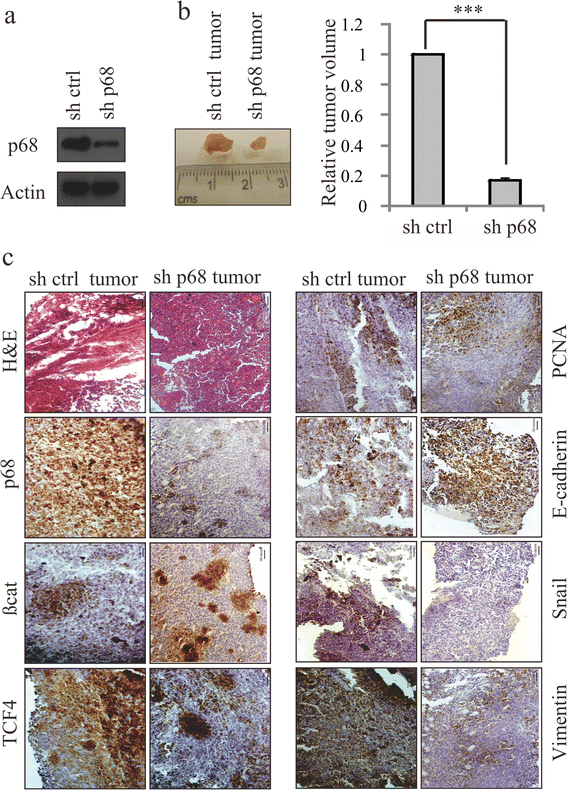

Supplement: Supplementary file 15 — Authors’ original file for figure 7 [file 13058_2014_496_MOESM15_ESM.gif]

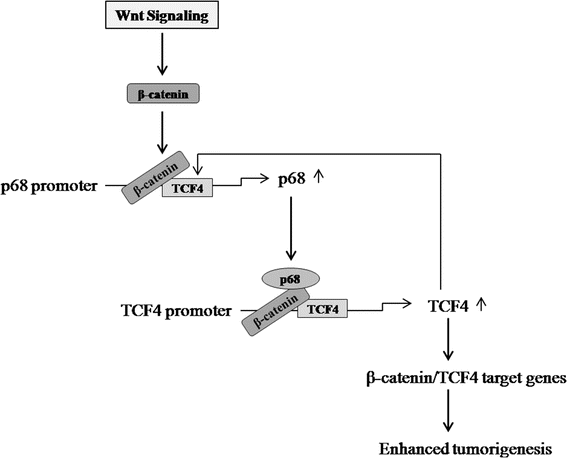

Supplement: Supplementary file 16 — Authors’ original file for figure 8 [file 13058_2014_496_MOESM16_ESM.gif]

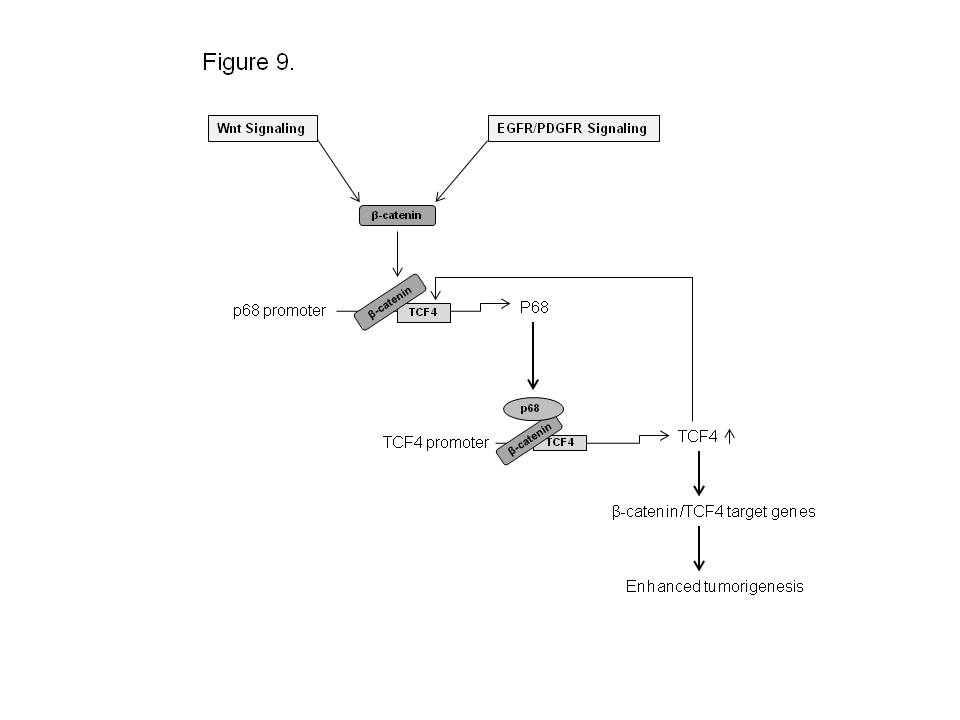

Supplement: Supplementary file 17 — Authors’ original file for figure 9 [file 13058_2014_496_MOESM17_ESM.jpeg]
